# *Streptococcus suis* Serotype 2 Infection Impairs Interleukin-12 Production and the MHC-II-Restricted Antigen Presentation Capacity of Dendritic Cells

**DOI:** 10.3389/fimmu.2018.01199

**Published:** 2018-05-30

**Authors:** Corinne Letendre, Jean-Philippe Auger, Paul Lemire, Tristan Galbas, Marcelo Gottschalk, Jacques Thibodeau, Mariela Segura

**Affiliations:** ^1^Laboratory of Immunology, Faculty of Veterinary Medicine, University of Montreal, Saint-Hyacinthe, QC, Canada; ^2^Laboratory of Research on Streptococcus suis, Faculty of Veterinary Medicine, University of Montreal, Saint-Hyacinthe, QC, Canada; ^3^Laboratory of Molecular Immunology, Department of Microbiology, Infectiology and Immunology, University of Montreal, Montreal, QC, Canada

**Keywords:** *Streptococcus suis*, inflammation, dendritic cells, T cells, antigen presentation, MHC-II, Th1 response, Interleukin-12

## Abstract

*Streptococcus suis* is an important swine pathogen and emerging zoonotic agent. Encapsulated strains of *S. suis* modulate dendritic cell (DC) functions, leading to poorly activated CD4^+^ T cells. However, the antigen presentation ability of *S. suis*-stimulated DCs has not been investigated yet. In this work, we aimed to characterize the antigen presentation profiles of *S. suis-*stimulated DCs, both *in vitro* and *in vivo*. Upon direct activation *in vitro, S. suis-*stimulated murine bone marrow-derived DCs (bmDCs) preserved their antigen capture/processing capacities. However, they showed delayed kinetics of MHC-II expression compared to lipopolysaccharide-stimulated bmDCs. Meanwhile, splenic DCs from infected mice exhibited a compromised MHC-II expression, despite an appropriate expression of maturation markers. To identify potential interfering mechanisms, Class II Major Histocompatibility Complex Transactivator (CIITA) and membrane-associated RING-CH (MARCH)1/8 transcription were studied. *S. suis-*stimulated DCs maintained low levels of *CIITA* at early time points, both *in vitro* and *in vivo*, which could limit their ability to increase MHC-II synthesis. *S. suis*-stimulated DCs also displayed sustained/upregulated levels of *MARCH1/8*, thus possibly leading to MHC-II lysosomal degradation. The bacterial capsular polysaccharide played a partial role in this modulation. Finally, interleukin (IL)-12p70 production was inhibited in splenic DCs from infected mice, a profile compatible with DC indirect activation by pro-inflammatory compounds. Consequently, these cells induced lower levels of IL-2 and TNF-α in an antigen-specific CD4^+^ T cell presentation assay and blunted T cell CD25 expression. It remains unclear at this stage whether these phenotypical and transcriptional modulations observed in response to *S. suis* in *in vivo* infections are part of a bacterial immune evasion strategy or rather a feature common to systemic inflammatory response-inducing agents. However, it appears that the MHC-II-restricted antigen presentation and Th1-polarizing cytokine production capacities of DCs are impaired during *S. suis* infection. This study highlights the potential consequences of inflammation on the type and magnitude of the immune response elicited by a pathogen.

## Introduction

*Streptococcus suis* is one of the most important bacterial pathogens in pigs causing meningitis, septicemia, and sudden death (1). It is responsible for major economic losses to the swine industry worldwide, and yet there is currently no real effective vaccine available to control infections caused by this bacterium (2). *S. suis* is also an emerging zoonotic agent that can cause meningitis and septicemia. High mortality rates have been observed in humans, particularly in cases of streptococcal toxic shock-like syndrome in Asia (1). Similarly, mice infected with *S. suis* have been shown to develop a strong systemic inflammatory response within 6 h post infection, and septicemia leading to death within 48 h (3–5). *S. suis* is an encapsulated bacterium, and a total of 35 serotypes have been defined based on the antigenicity of their capsular polysaccharides (CPS) (2). Serotype 2 is the most virulent for both pigs and humans, and most studies have been performed with this serotype (1). *S. suis* possesses several virulence factors (6), among which the CPS is clearly critical for the pathogenesis of *S. suis* infections (7).

Dendritic cells (DCs) are the most potent antigen-presenting cells (APCs); they connect innate and adaptive immunity (8, 9). During an infection, DC maturation can be initiated indirectly by inflammatory mediators released by innate immune cells [indirectly activated mature DCs (indir-mDCs)] or through direct contact with the pathogen [directly activated mature DCs (dir-mDCs)] (10). In both instances, DC maturation is characterized by the expression of cell surface molecules, particularly the MHC class II (MHC-II) molecules and costimulatory molecules, such as CD86 (10, 11). DCs that have captured a pathogen then process it and load its derived antigenic peptides on their MHC-II molecules (12), forming peptide-MHC-II complexes (pMHC-II) that will be exported from the endosomal peptide-loading compartments to the cell surface (12, 13). The whole process is usually complete within 1–3 h (14). These pMHC-II will then be recognized by an antigen-specific T cell receptor (TCR) (15, 16). Specific pMHC-II recognition is the first signal for CD4^+^ T cell activation and is essential for the induction of the adaptive response (17). The second signal determines the ability of the antigen-specific CD4^+^ T cell to expand and involves binding of the costimulatory molecules on the naïve T cell (17, 18). Finally, the third signal for CD4^+^ T cell activation is conveyed by DC-derived cytokines that will induce T cell polarization toward different CD4^+^ T helper lineages with distinct effector functions (18, 19). Host protection against infections caused by *S. suis* is mediated primarily by opsonophagocytosis, a process favored by type 1 IgG subclasses. These antibody subclasses with a high protective potential are mainly associated with Th1-type immune responses (2). Interleukin (IL)-12 is known as the primary cytokine for the differentiation of the Th1 subset (20). However, indir-mDCs do not secrete IL-12 in situations where dir-mDCs do and are thus unable to induce functional T cell responses (20, 21).

Different antigenic peptides can be loaded either on newly synthesized or on recycling MHC-II molecules (14). MHC-II transcription is tightly regulated by the Class II Major Histocompatibility Complex Transactivator (CIITA); this master regulator induces *de novo* transcription of MHC-II genes (13, 21). Upon exposure to a Toll-like receptor (TLR) ligand, a transient increase in MHC-II synthesis has been observed as early as 1 h after challenge (14). However, CIITA transcription (and thus the ensuing MHC-II synthesis) is severely reduced within hours (22, 23), as well as the uptake of new antigens for processing (8, 22). Independently from CIITA control, MHC-II expression also undergoes regulation at the protein level (13). The trafficking of MHC-II molecules and their cell surface expression are regulated, among other mechanisms, *via* ubiquitination by ubiquitin ligases of the membrane-associated RING-CH (MARCH) family, particularly MARCH1 and MARCH8 (11, 13, 15). In fact, ubiquitination by MARCH1 of the transmembrane glycoproteins MHC-II and CD86 is known to lead to lysosomal degradation of these molecules in immature DCs (11). However, MARCH1/8 expression is downregulated in dir-mDCs (11, 21, 24). It has been suggested that while MARCH1 activity allows the turnover of various pMHC-II in immature DCs, termination of MARCH1 expression in dir-mDCs would considerably prolong the half-life of pMHC-II and CD86 and enhance the stability of pMHC-II derived from the activating pathogen (11). Such regulation processes would allow the DC to present large and stable amounts of relevant pMHC-II, thereby increasing its ability to activate an antigen-specific CD4^+^ T cell in an efficient manner (22, 23, 25). By contrast, indir-mDCs retain their capacity to present new antigens and have a high pMHC-II turnover rate (thus reducing the stability of relevant pMHC-II derived from the pathogen) as they do not downregulate MARCH1 synthesis (26, 27).

*Streptococcus suis* recognition by DCs has been reported to occur essentially through TLR2 (28). Encapsulated strains of *S. suis* have been shown to modulate DC functions in a variety of ways. First of all, these strains exhibit anti-phagocytic properties in murine, porcine, and human DCs (7, 9, 29, 30). They also modulate the expression of the maturation markers CD80/CD86 and MHC-II in murine and porcine DCs (9, 29) and CD83/CD86 in human DCs (30). Moreover, encapsulated strains are known to impair cytokine production/release by DCs from all three species; the CPS most probably hinders the recognition of immunogenic cell wall components (9, 29, 30). However, the antigen presentation ability of *S. suis*-stimulated DCs has never been investigated thoroughly. Interference of *S. suis* with the signals required for antigen presentation in DCs could have profound consequences on the development of the ensuing adaptive response (31). This could account, at least in part, for the weak CD4^+^ T cell activation (32), the low primary and memory humoral responses observed in both mice and pigs (2, 33), as well as for some of the difficulties experienced in developing an effective vaccine to control *S. suis* disease in swine (2). Finally, the low antibody titers obtained against a bystander antigen [ovalbumin (OVA)] in *S. suis*-preinfected mice (32) suggest that the antigen presentation machinery is altered. It is hypothesized here that *S. suis* interferes with the ability of DCs to present antigens to CD4^+^ T cells *via* the MHC-II pathway and thus compromises the development of an efficient adaptive immune response. The purpose of the present study was to investigate in an *in vitro, in vivo*, and *ex vivo* mouse model the signals involved in antigen presentation, from antigen capture and processing to T cell-polarizing cytokine production, in *S. suis*-stimulated DCs. A non-encapsulated *S. suis* serotype 2 mutant was also included in the study to dissect the role of the CPS regarding the MHC-II pathway.

## Materials and Methods

### Ethics Statement

This study was carried out in accordance with the recommendations of the guidelines and policies of the Canadian Council on Animal Care and the principles set forth in the Guide for the Care and Use of Laboratory Animals. The protocols and procedures were approved by the Animal Welfare Committee of the University of Montreal (protocol number rech-1399).

### Bacterial Strains

*Streptococcus suis* serotype 2 virulent strain P1/7 and its isogenic non-encapsulated mutant strain Δ*cpsF* (29) were used. *S. suis* strains were grown on sheep blood agar plates at 37°C for 18 h, and isolated colonies were used as inocula for Todd–Hewitt Broth (THB) (Becton Dickinson, Mississauga, ON, Canada). For *in vitro* experiments, 5 ml of inoculated THB was incubated for 16 h at 37°C with agitation; working cultures were then obtained by inoculating 300 µl of these cultures in 10 ml of THB and incubating for 5 h at 37°C with agitation. For the *in vivo* experiment, 5 ml of inoculated THB was incubated for 8 h at 37°C with agitation; working cultures were obtained by inoculating 10 µl of a 10^−3^ dilution of these cultures in 30 ml of THB and incubating for 16 h at 37°C with agitation. Both bacterial growth protocols were standardized to obtain late-logarithmic bacteria timely synchronized for *in vitro* or *in vivo* infections. Bacteria were washed twice in phosphate-buffered saline (PBS), pH 7.3, and appropriately diluted in complete cell culture medium for *in vitro* assays or THB for the *in vivo* experiment. The number of CFU/ml in the final suspension was determined by plating samples onto THB agar using an Autoplate 4000 Automated Spiral Plater (Spiral Biotech, Norwood, MA).

### Mice and CD4^+^ T Cell Hybridoma

C57BL/6 female mice were purchased from Charles River Laboratories (Wilmington, MA, USA). Animals were used at 5–6 weeks of age. The BO97.10 CD4^+^ T cell hybridoma specific for the OVA_323–339_ peptide on I-A^b^ (a kind gift from M. Desjardins) was maintained in Kappler–Marrack complete medium, as previously described (34).

### Reagents

Anti-mouse antibodies (BD Pharmingen, Mississauga, ON, Canada; unless otherwise noted) used for FACS analysis were as follows: FITC-conjugated anti-CD25 (7D4), Alexa488-conjugated anti-CD11c (N418; BioLegend, San Diego, CA, USA), PE-conjugated anti-I-A^b^ (AF6-120.1) and anti-CD86 (GL1), PE/Cy5-conjugated anti-CD3ε (145-2C11) and anti-CD11c (N418; BioLegend), and APC-conjugated IL-12p40 (C15.6) and anti-CD11c (HL3).

### Generation of Mouse Bone Marrow-Derived Dendritic Cells (bmDCs)

Cells were generated from naïve mice as previously described (28). Briefly, bone marrow was removed from femurs and tibiae. After red blood cell lysis (eBioScience, San Diego, CA, USA), the total bone marrow cells (2.5 × 10^5^ cells/ml) were cultured in RPMI 1640 supplemented with 5% heat-inactivated FBS, 10 mM HEPES, 20 µg/ml gentamycin, 100 U/ml penicillin–streptomycin, 2 mM l glutamine and 50 µM 2-ME (Gibco, Invitrogen, Burlington, ON, Canada). Complete medium was complemented with 20% GM-CSF from a mouse GM-CSF-transfected cell line (Ag8653) as a source of GM-CSF (35). Cells were cultured for 7 days at 37°C in a 5% CO_2_ incubator, and fresh medium was added on days 3 and 5. On day 7, clusters were harvested and subcultured overnight to remove adherent cells. Non-adherent cells were harvested on day 8, washed, and used as immature DCs for the studies in complete medium containing 5% GM-CSF. Cell purity routinely comprised 86–90% CD11c^high^, as determined by FACS analysis and as previously reported (28).

### *In Vitro* Analysis of OVA Uptake and Processing by bmDCs

BODIPY dye (DQ-OVA, Molecular Probes, Invitrogen), a self-quenching molecule that is degraded into peptides that exhibit a bright, photostable fluorescence after uptake and intracellular processing, was used as described before to determine cellular internalization and processing of OVA by DCs (36). Briefly, bmDCs were suspended at 5 × 10^6^ cells/ml in RPMI complete medium (without antibiotics) and incubated with *S. suis* strain P1/7 (5 × 10^7^ CFU/ml; initial MOI: 10) for 45 min. Then, bmDCs were washed and incubated with 50 µg/ml of OVA labeled with BODIPY dye or with non-labeled OVA for 15 min at 37°C in complete medium. After three washing steps with cold PBS to remove non-internalized OVA, cells were suspended in PBS with 5% FBS and incubated at 37°C (time 0). The processing of OVA into peptides after internalization was assayed at different times ranging from 0 to 60 min by FACS. The culture conditions and concentrations of DQ-OVA were determined based on pretrials (data not shown). Non-infected cells served as negative control.

### *In Vitro* bmDCs Stimulation Assay

A total of 1 × 10^6^ cells in RPMI complete medium (without antibiotics) were seeded into 24-well flat-bottomed plates (500 µl/well). Cells were allowed to rest for 1 h at 37°C with 5% CO_2_. Afterward, cells were stimulated in technical duplicates for each condition with *S. suis* strains P1/7 and Δ*cpsF* (1 × 10^6^ CFU; initial MOI:1; final volume 1 ml). Conditions used were based on those already published (28). Cells were harvested after the desired incubation time for FACS and RT-qPCR analysis. Unstimulated cells served as negative control at each time point, while cells treated with the TLR4 ligand lipopolysaccharide (LPS) at 1 µg/ml [from *Escherichia coli* 0127:B8 (Sigma-Aldrich, Oakville, ON, Canada)] were used as positive control at each time point.

### Isolation of Splenic DCs From Naïve Mice and Stimulation *In Vitro*

Spleen cells were obtained from a pool of 10 naïve mice. Spleens were harvested, perfused with RPMI complete medium (Gibco), and pressed gently through a sterile fine wire mesh. After red blood cells lysis (eBioscience), total splenocytes were suspended in 2 mM EDTA-PBS solution and separated using Lympholyte-M density gradient (Cedarlane Labs, Burlington, ON, Canada). Low-density cells at the interphase were isolated and then further FACS-purified for CD11c^+^ (APC-conjugated) with the FACSAria Fusion flow sorter (BD Biosciences, San Jose, CA, USA) using “low-recovery high-purity” sorting settings. A purity of >98% was obtained. Cells were allowed to rest for 1 h at 37°C with 5% CO_2_. Afterward, cells were stimulated with *S. suis* strains P1/7 and Δ*cpsF* (initial MOI:1) or LPS at 1 µg/ml for 2, 4, and 18 h.

### *In Vivo* Infection Model and Isolation of Splenic DCs

On the day of the experiment, mice were injected intraperitoneally with 1 ml of the bacterial suspension (5 × 10^7^ CFU of *S. suis* strain P1/7) or sterile vehicle solution (THB), as previously standardized in our laboratory (3). The use of 5 × 10^7^ CFU was the optimal dose to induce a positive bacteremia and elicit clinical signs of disease and a strong inflammatory response within 6 h. Spleens from naïve and infected mice were harvested 3 or 6 h post infection and processed individually (see the number of animals in figure legends). Infected mice showing clinical signs of septic disease (e.g., ruffled coat, prostration, and depression) were selected for the experiments. At the time of euthanasia, blood was collected by terminal cardiac puncture and bacteremia (the number of CFU/ml) was determined by plating samples onto THB agar using an Automated Spiral Plater. Infected mice had a positive bacteremia (~1–3 × 10^8^ CFU/ml of blood) as previously reported (32, 37). For the purification of splenic DCs, spleens (from either naïve or infected mice) were harvested and total splenocytes were separated using Lympholyte-M density gradient as described above. Low-density cells at the interphase were purified by MACS positive selection using CD11c MicroBeads and MS columns (Miltenyi Biotec, Auburn, CA, USA) as per the manufacturer’s recommendations. The enriched CD11c^+^ cells had >86–90% purity and similar yield for both naïve and infected mice by FACS analysis using CD11c antibody (Figure 2A).

### *Ex Vivo* Splenic DC Stimulation Assay

Splenic DCs from naïve or infected mice were isolated at 6 h post infection as described above. A total of 5 × 10^5^ cells in RPMI complete medium with antibiotics (100 µg/ml gentamycin) were seeded into 48-well flat-bottomed plates (250 µl/well). Cells were allowed to rest for 1 h at 37°C with 5% CO_2_. Afterward, cells were stimulated either with CpG oligodeoxynucleotides at 1 µM (ODN 1826; Invivogen, San Diego, CA, USA) or LPS at 1 µg/ml (final volume 500 µl). Plates were incubated for 24 h, before supernatant harvesting for cytokine quantification. Unstimulated cells served as negative controls or basal expression.

### *Ex Vivo* Antigen Presentation Assay

Splenic DCs from naïve or infected mice were isolated at 6 h post infection as described above. A suspension of 1 × 10^5^ cells/ml in Kappler–Marrack complete medium with antibiotics (100 µg/ml gentamycin) were seeded into 48-well flat-bottomed plates (250 µl/well). Cells were allowed to rest for 1 h at 37°C with 5% CO_2_. Afterward, CD4^+^ T cells (BO97.10) were added to the wells (DC:T cell ratio of 1:3, final volume 500 µl) in Kappler–Marrack complete medium containing OVA (500 µg/ml; grade VII, Sigma). Cocultures incubated with medium alone served as negative controls, while cocultures treated with LPS (1 µg/ml) and OVA served as positive controls. Coculture plates were incubated for 24 h. The supernatant was harvested for ELISA testing, while cells were collected for FACS analysis. Single cell cultures (either DCs alone or T cells alone) were also included as controls. Culture conditions were based on *in vitro* pretrials (Figure S1 in Supplementary Material and data not shown).

### FACS Analysis

To determine cellular internalization and processing of OVA by DCs, FACS was performed after each incubation time using a Cell Lab Quanta™ SC MultiPlate Loader instrument (Beckman Colter, Mississauga, ON, Canada). 20,000 gated events were acquired per sample. Data are expressed as the increase in the mean fluorescence intensity (MFI) over time and were analyzed using Cell Lab Quanta Collection/Analysis software. For evaluation of MHC-II and CD86 cell surface expression *in vitro*, bmDCs were harvested at each time point, washed, and fixed (eBioScience). Cells were then treated with an FcR-blocking reagent (Fc*γ*III/II Rc Ab, BD Pharmingen) for 15 min on ice. Cells were stained with PE/Cy5-conjugated anti-CD11c (45 min on ice), then washed and stained with either PE-conjugated anti-CD86 or anti-MHC-II I-A^b^ (45 min on ice). After washing steps, cells were suspended in sorting buffer for FACS analysis. Splenic DCs from the *in vivo* experiment were similarly treated for the evaluation of MHC-II and CD86 expression. *In vitro-*stimulated splenic DCs were stained only with PE-conjugated anti-CD86 or anti-MHC-II I-A^b^ as they had already been stained with APC-conjugated anti-CD11c for FACS sorting. For IL-12p40 intracellular staining, splenic DCs were surface stained with Alexa488-conjugated anti-CD11c (45 min on ice), fixed and permeabilized using the IC Fixation/Permeabilization kit (eBioScience) as per the manufacturer’s recommendation. Following fixation and permeabilization, cells were stained (20 min at room temperature) with APC-conjugated anti-IL-12p40. For evaluation of CD25 expression on DC–T cell cocultures, cells were harvested, washed, blocked, and surface stained with FITC-conjugated anti-CD25 (45 min on ice), before washing steps and staining with PE/Cy5-conjugated anti-CD3ε or APC-conjugated anti-CD11c. For the *in vitro* experiment with splenic DCs, flow cytometry analysis was performed with the FACSAria™ Fusion flow sorter (BD Biosciences) and data were analyzed using the FACSDiva software. For all other experiments, flow cytometry was performed using a BD Accuri™ C6 cytometer (BD Biosciences, Mississauga, ON, Canada). At least 30,000 gated events were acquired per sample, and data analysis was performed using BD Accuri™ C6 software. Quadrants were drawn based on single stain and isotype controls and were plotted on logarithmic scales.

### Quantitative Real-Time PCR

Bone marrow-derived dendritic cells and splenic DCs from the *in vitro* and *in vivo* experiments were washed, transferred in QIAzol (1 ml/10^6^ cells; Qiagen, Mississauga, ON, Canada), and maintained at −80°C after each time point. The total cellular RNA was later extracted and quantified by spectrophotometry (Nanodrop ND-1000). Total RNA (500 ng) was reverse-transcribed with the QuantiTect^®^ Reverse Transcription kit (Qiagen) as per the manufacturer’s recommendations. The cDNA was amplified using the SsoFast™ EvaGreen^®^ Supermix kit (Bio-Rad, Hercules, CA, USA). A real-time thermal cycler CFX96 (Bio-Rad) was used for amplification of target cDNA, and quantitation of differences between the different groups was calculated using the 2^−ΔΔ^*^C^*^t^ method. Peptidylprolyl isomerase A (PPIA) was used as the normalizing gene to compensate for potential differences in cDNA amounts. The unstimulated DCs were used as the calibrator reference in the analysis. Each sample was run in triplicates and a no-template control without cDNA was run for every primer set. The primers used for amplification of the different target cDNA (Integrated DNA Technologies, Coralville, IA) are as follows: CIITA, forward 5′-AACCTGCGCTGACCTCCCGTGTAA-3′ and reverse 5′-GCTCCCTTTCCTGGCTCTTGTTGC-3′; MARCH1, forward 5′-CAGATGACCACGAGCGAAAG-3′ and reverse 5′-CCAATAGCCACCACAACCAG-3′; MARCH8, 5′-TGGCTTCATGTTGTTCCCTTTATTTC-3′ and reverse 5′-CAGCCGTGCCTTGCCAGTC-3′; PPIA, 5′-TGCTGGACCAAACACAAACGGTTC-3′ and reverse 5′-CAAAGACCACATGCTTGCCATCCA-3′.

### Cytokine Quantification by ELISA

Interleukin-12p70 and IL-10 levels in *ex vivo* supernatants, as well as IL-2, TNF-α, IFN-γ, and IL-10 levels in DC–T cell coculture supernatants, were measured by sandwich ELISA using pair-matched antibodies (R&D Systems, Minneapolis, MN, USA) as per the manufacturer’s recommendations. Twofold dilutions of recombinant murine cytokines were used to generate the standard curves. Sample dilutions giving optical density readings in the linear portion of the appropriate standard curve were used to quantify the levels of the cytokine. Absorbance was measured at 450 nm.

### Cytokine and Chemokine Quantification by Luminex

Interleukin-6, G-CSF, CCL2 (MCP-1), CCL3 (MIP-1α), CCL4 (MIP-1β), CCL5 (RANTES), CXCL2 (MIP-2), and CXCL9 (MIG) levels in *ex vivo* supernatants were determined using a liquid multiarray system (Bio-Plex Pro™) according to the manufacturer’s instructions. Commercial multiplex-coated beads (custom-made cytokine panel), biotinylated Abs, and Beadlyte microtiter 96-well filter plates were obtained from Bio-Rad. Data were collected with Bio-Plex Manager™ software and analyzed with Bio-Plex^®^ MAGPIX system. Standard curves for each cytokine and chemokine were obtained using the reference concentrations supplied by the manufacturer.

### Statistical Analysis

Data for the *in vitro* kinetic experiments were analyzed for significance using a two-way analysis of variance (ANOVA). For the *in vivo* transcription profile of splenic DCs and the coculture experiments, a one-way ANOVA was used. All pairwise comparisons were run, and *P*-values were Bonferroni-adjusted. The FACS data for the *in vivo* experiments and data for the *ex vivo* stimulation experiments were analyzed using Student’s unpaired *t-*test. A *P* < 0.05 was considered as statistically significant. Data are expressed as mean ± SEM. All statistical analyses were performed using the IBM SPSS Statistics software (v. 24).

## Results

### *S. suis* Does Not Interfere With Soluble Antigen Uptake and Processing in bmDCs

The ability of *S. suis-*stimulated bmDCs to capture and process a reporter antigen linked to a self-quenching molecule, DQ-OVA, was first investigated. Although bmDCs preincubated with *S. suis* tended to express lower MFI of DQ-OVA than unstimulated cells at all time points, there was no statistically significant difference (Figure S2 in Supplementary Material). Similar data were obtained when using different DQ-OVA doses, bacterial MOI, and incubation times (data not shown). *S. suis-*stimulated bmDCs thus appear to have intact soluble antigen capture and processing capacities.

### *In Vitro* Stimulation of bmDCs With the Encapsulated Strain of *S. suis* Induces an Enhanced Expression of MHC-II, but With a Delayed Kinetics

**Figure 1:**
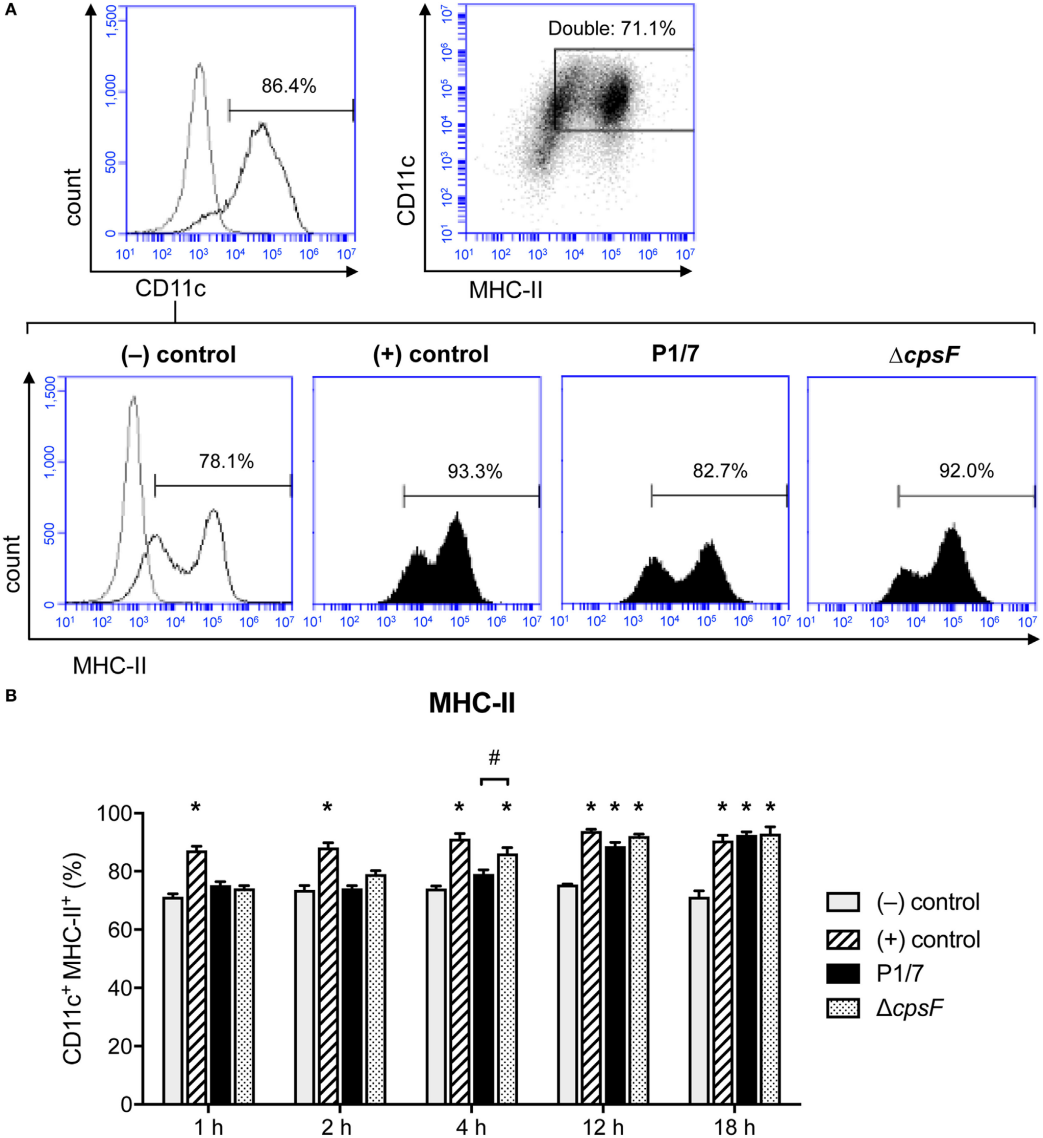
Bone marrow-derived dendritic cells (BmDCs) enhance their MHC-II cell surface expression with a delayed kinetics upon stimulation with *Streptococcus suis* wild-type (WT) strain. Cells were stimulated for 1, 2, 4, 12, or 18 h with *S. suis* WT strain P1/7 or the non-encapsulated mutant Δ*cpsF* (initial MOI:1) in technical duplicates. Unstimulated cells served as negative (−) control for basal expression at each time point. Cells stimulated with LPS (1 µg/ml) were used as positive (+) control. Cells were harvested and fixed after each incubation time. Once the last incubation time was over, cells were surface stained for CD11c and MHC-II and analyzed by FACS. Events are gated on CD11c^+^ cells. **(A)** Representative histograms have been selected for this figure (at time = 4 h). The gray lines are isotype controls. At least 30,000 gated events were acquired per sample and data analysis was performed using BD Accuri™ C6 software. Quadrants were drawn based on PE/Cy5- and PE-control stains and plotted on logarithmic scales, a representative dot plot is displayed. **(B)** Data are expressed as mean ± SEM (% of positive cells) and are from three independent experiments. *P < 0.05 indicates a statistically significant difference compared to (−) control cells. ^#^*P* < 0.05 indicates a statistically significant difference between P1/7-stimulated bmDCs and Δ*cpsF*-stimulated bmDCs.

MHC-II cell surface expression (T cell activation signal 1) was investigated in naïve and *S. suis*-stimulated bmDCs at 1, 2, 4, 12, and 18 h after stimulation. Moreover, as *S. suis* is an encapsulated bacterium, the impact of CPS on MHC-II expression was evaluated by comparing the wild-type (WT) strain P1/7 with its non-encapsulated mutant, Δ*cpsF*. LPS-treated bmDCs served as positive controls for the expected kinetics of MHC-II expression in bmDCs responding to a TLR ligand (dir-mDCs). While LPS-treated bmDCs significantly increased their MHC-II expression within 1 h, *S. suis*-stimulated cells only reached such high percentages of MHC-II^+^ cells at late time points (Figure 1). Overall, the total MFI levels were similar among treatments, but a similar delay in the increase of MFI levels was observed in *S. suis*-stimulated cells when considering the MHC^low^ subpopulation (P1, see Figure S3 in Supplementary Material). In fact, bmDCs stimulated with the WT strain showed no significant increase of MHC-II expression at early time points, in contrast to Δ*cpsF*-stimulated cells. A statistically significant difference was observed between the two strains at 4 h, thus suggesting that the presence of the CPS on *S. suis* plays a partial role in the delayed maturation process underwent by *S. suis*-stimulated bmDCs.

### *S. suis* Induces Splenic DC Maturation *In Vivo* but With a Low Intensity of MHC-II Expression on the Cell Surface

**Figure 2:**
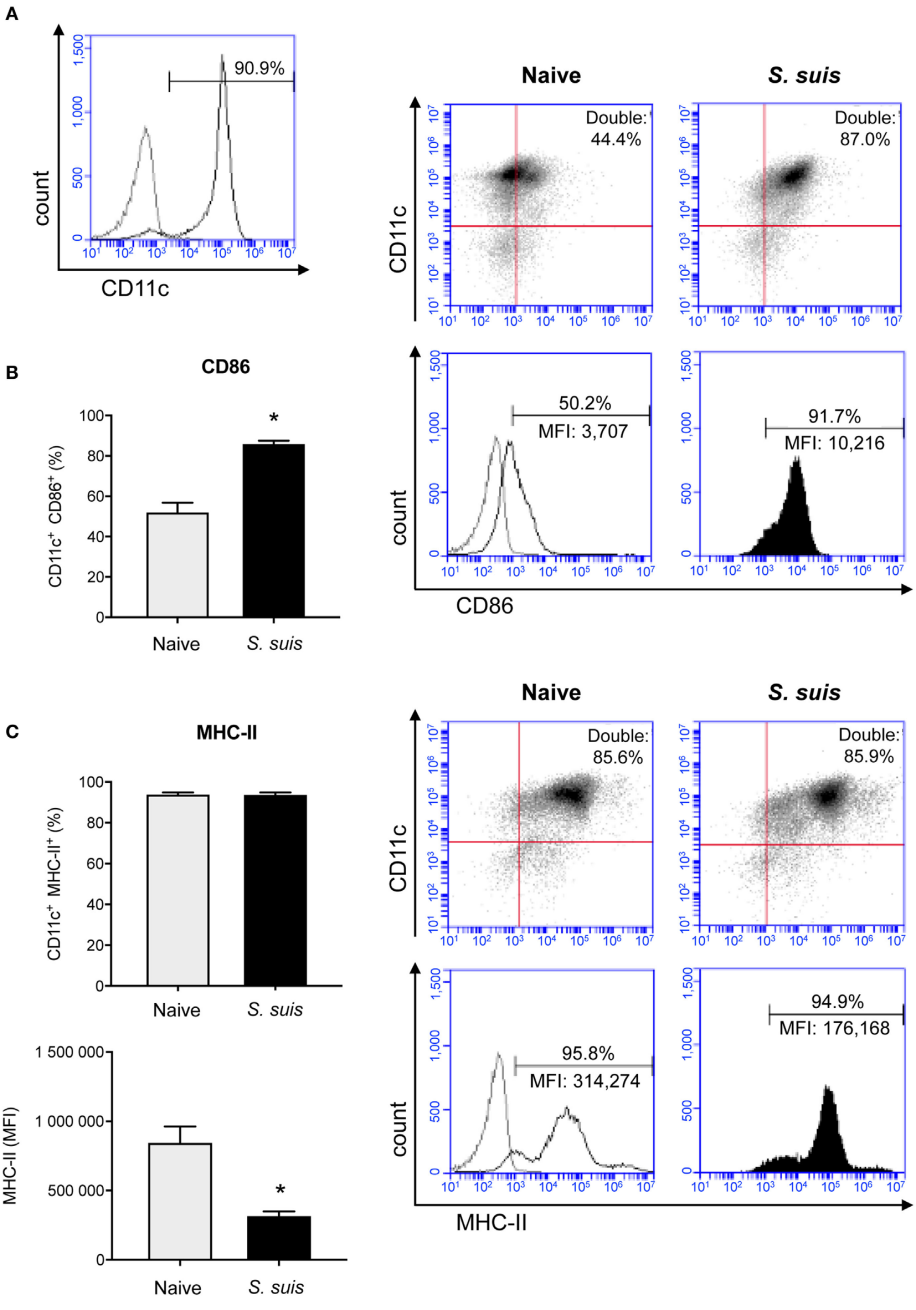
Splenic dendritic cells (DCs) become phenotypically mature but show a reduction in their MHC-II expression levels during *Streptococcus suis in vivo* infection. C57BL/6 mice were injected intraperitoneally with 5 × 10^7^ CFU of *S. suis* wild-type (WT) strain P1/7. Spleens from mock-infected (naïve) and infected mice were harvested 6 h after infection (*n* = 2–3 per group × 2 independent experiments). Splenic DCs were purified by MACS positive selection and cells were surface stained for FACS analysis. CD11c purity is shown in **(A)**. The expression of **(B)** CD86 and **(C)** MHC-II was evaluated. Data are expressed as mean ± SEM (of % double positive cells or of mean fluorescence intensity (MFI). In the latter case, events are gated on CD11c^+^ cells). Representative dot plots and histograms have been selected for this figure. The gray lines on the histograms are isotype controls. 30,000 events were acquired per sample and data analysis was performed using BD Accuri™ C6 software. Quadrants were drawn based on PE/Cy5- and PE-control stains and plotted on logarithmic scales. *P < 0.05 indicates a statistically significant difference compared to naïve cells.

MHC-II expression was investigated in purified splenic DCs in an *in vivo* mouse model. Unfortunately, as non-encapsulated *S. suis* mutants are rapidly cleared from circulation (6), *in vivo* investigation of the role of this virulence factor was impossible and the experiment had to be conducted with the WT strain only. Splenic DCs have never been studied in the context of *S. suis* infection; we thus evaluated the maturation profile of these cells during an acute systemic infection, along with their MHC-II expression. Splenic DCs derived from *S. suis-*infected mice showed an increased expression (at both % and MFI levels) of the DC maturation marker CD86 (T cell activation signal 2) compared to naïve mice (Figure 2B). This suggests that the initiation of the DC maturation process occurs quickly in splenic DCs during a systemic infection with *S. suis*. Moreover, the percentage of splenic DCs expressing MHC-II molecules on their cell surface remained elevated after infection and was therefore in agreement with this DC maturation profile (Figure 2C). However, DCs from *S. suis-*infected mice showed a lower MFI. This observation could imply that the total number of MHC-II molecules on the surface of each MHC-II^+^ cell was reduced; in spite of the initiation of the DC maturation process, *S. suis* could downregulate the expression of MHC-II molecules on splenic DCs *in vivo*.

### BmDCs and Splenic DCs Stimulated *In Vitro* With *S. suis* Have Different Maturation Profiles Compared to Splenic DCs From Infected Mice

**Figure 3:**
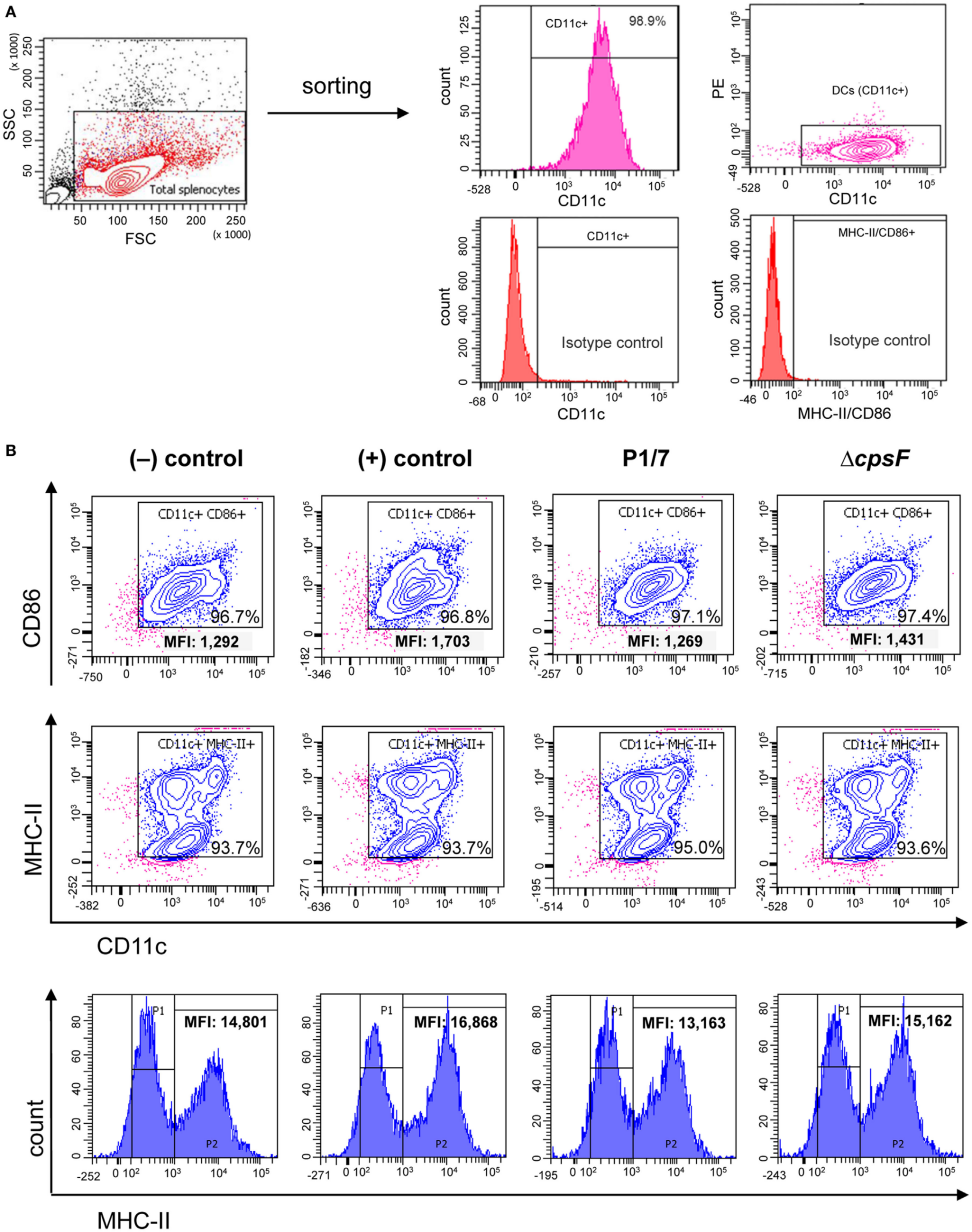
Naïve splenic dendritic cells (DCs) show low intensity of MHC-II expression upon *in vitro* stimulation with the encapsulated strain of *Streptococcus suis*. Spleen cells from a pool of 10 naïve C57BL/6 mice were FACS-purified and CD11c^+^ cells (APC-conjugated anti-CD11c) were sorted with the BD FACSAria™ Fusion flow sorter as illustrated in **(A)**. **(B)** Purified cells were stimulated for 2, 4, or 18 h with *S. suis* wild-type (WT) strain P1/7 or the non-encapsulated mutant Δ*cpsF* (initial MOI:1). Unstimulated cells served as negative (−) control for basal expression at each time point. Cells stimulated with lipopolysaccharide (LPS) (1 µg/ml) were used as positive (+) control. Cells were harvested and surfaced stained for CD86 or MHC-II. Representative density plots have been selected for this figure (at time = 18 h). Histograms of MHC-II expression (gated on CD11c^+^ cells) have also been included to illustrate variations in the mean fluorescence intensity (MFI) of the MHC-II^high^ population (P2, at time = 18 h). Data analysis was performed using FACSDiva software. Density plots were drawn based on APC- and PE-control stains and plotted on logarithmic scales.

As bmDCs and splenic DCs from infected mice were found to differ in their patterns of MHC-II expression levels, we investigated whether these differences were inherent to the cell type or were rather due to complex interactions occurring *in vivo* (i.e., direct vs indirect activation). We stimulated naïve splenic DCs *in vitro* and evaluated their MHC-II expression kinetics in response to *S. suis*. All treatments yielded high percentages of MHC-II^+^ and CD86^+^ cells, but analysis of the MHC-II^high^ subpopulation revealed a consistently lower MHC-II MFI for WT *S. suis*-stimulated cells, as compared to cells stimulated with LPS or the non-encapsulated mutant (Figure 3; Figure S4 in Supplementary Material). This maturation profile is similar to those observed at short incubation times in bmDCs (Figure 1B). However, while bmDCs upregulate their MHC-II and CD86 expression at late time points [Figure 1; (29)], WT *S. suis*-stimulated splenic DCs still displayed low MHC-II MFI levels as late as 18 h (Figure 3B). Splenic DCs stimulated *in vitro* with the WT strain also maintained low CD86 MFI levels (Figure 3B), which contrasts with the maturation profile obtained in splenic DCs from infected mice (Figure 2B). These results suggest that while *S. suis*–DC interactions might partly depend on the cell origin, other complex modulations are also likely to be involved *in vivo*.

### *S. suis-*Stimulated DCs Maintain Low Transcriptional Levels of CIITA, Both *In Vitro* and *In Vivo*

**Figure 4:**
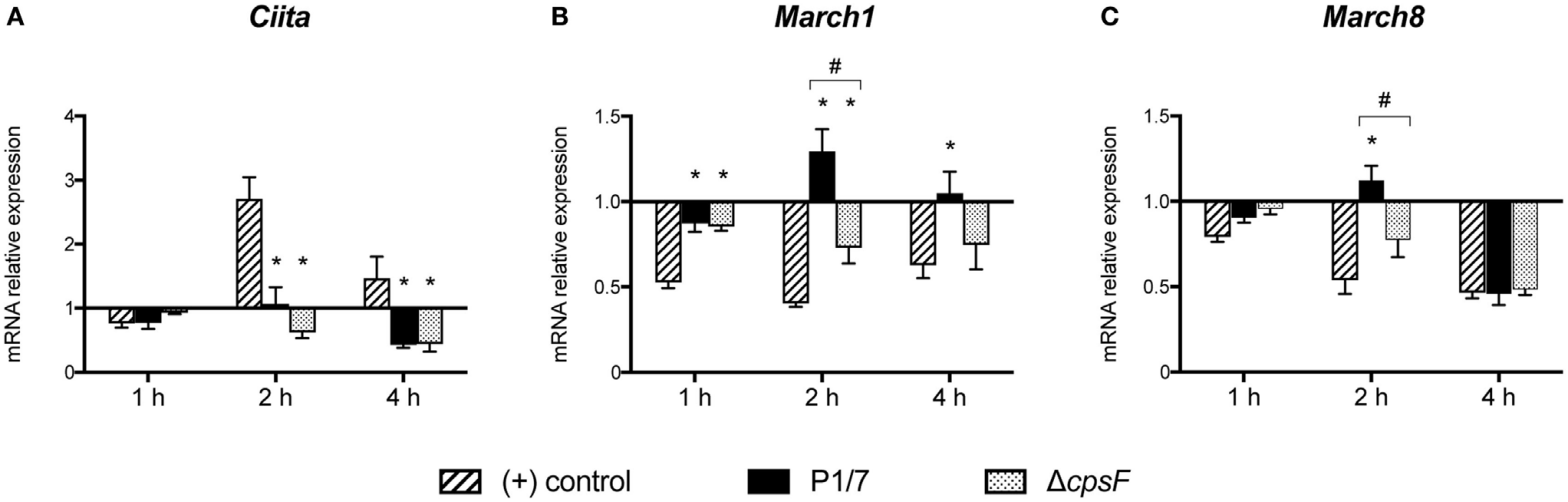
*Streptococcus suis*-stimulated bone marrow-derived dendritic cells (bmDCs) and directly activated bmDCs have distinct Class II Major Histocompatibility Complex Transactivator (CIITA), MARCH1 and MARCH8 transcriptional profiles. BmDCs were stimulated for 1, 2, or 4 h with *S. suis* wild-type (WT) strain P1/7 or the non-encapsulated mutant Δ*cpsF* (initial MOI:1) in technical duplicates. Total cellular RNA was extracted and analyzed by RT-qPCR for **(A)** *CIITA*, **(B)** *MARCH1*, and **(C)** *MARCH8* mRNA expression. Expression is illustrated as fold level over unstimulated cells [(−) control]. Cells stimulated with lipopolysaccharide (LPS) (1 µg/ml) served as positive (+) control. Data are expressed as mean ± SEM from four independent experiments. *P < 0.05 indicates a statistically significant difference compared to (+) control cells. ^#^*P* < 0.05 indicates a statistically significant difference between P1/7-stimulated bmDCs and Δ*cpsF*-stimulated bmDCs.

**Figure 5:**
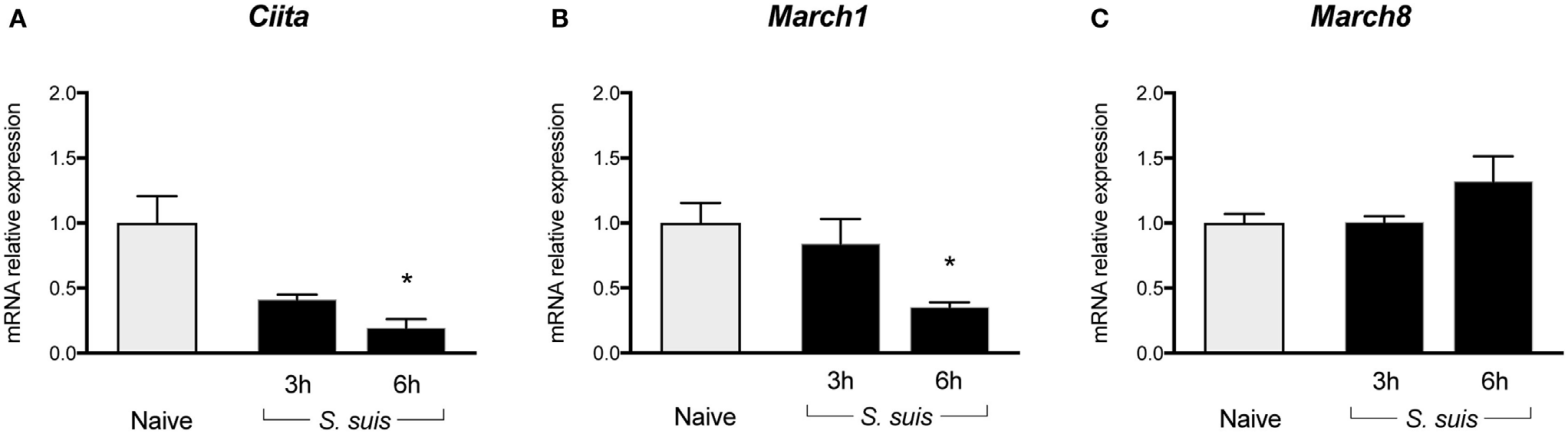
Splenic dendritic cells (DCs) show reduced transcription of Class II Major Histocompatibility Complex Transactivator (CIITA) and sustained/upregulated transcription of MARCH1 and MARCH8 during *Streptococcus suis* infection. C57BL/6 mice were injected intraperitoneally with 5 × 10^7^ CFU of *S. suis* wild-type (WT) strain P1/7. Spleens from mock-infected (naïve) and infected mice were harvested 3 or 6 h after infection (*n* = 2 to 3 per group × 2 independent experiments). Splenic DCs were purified by MACS positive selection, then total cellular RNA was extracted and analyzed by RT-qPCR for **(A)** *CIITA*, **(B)** *MARCH1*, and **(C)** *MARCH8* mRNA expression. Expression is illustrated as fold level as compared to expression in naïve mice. Data are expressed as mean ± SEM. *P < 0.05 indicates a statistically significant difference compared to naïve cells.

Given the key role of CIITA in *de novo* transcription of MHC-II genes and its potential implication in the modulation of MHC-II expression by *S. suis*, the transcriptional expression of CIITA was evaluated in *in vitro-*stimulated bmDCs as well as in splenic DCs derived from infected mice. *CIITA* expression was investigated in bmDCs stimulated with the WT strain and its non-encapsulated mutant for 1, 2, or 4 h. Interestingly, *S. suis*-stimulated bmDCs showed globally low levels of *CIITA* for both strains (Figure 4A). No significant difference was observed between the WT strain and its non-encapsulated mutant. By contrast, under our assay conditions, LPS-treated bmDCs showed *CIITA* upregulation at 2 and 4 h, as reported previously (23). The expression of *CIITA* mRNA was also investigated *in vivo* at 3 and 6 h after infection, with splenic DCs derived from *S. suis*-infected mice showing low levels of *CIITA* compared to naïve mice (Figure 5A). These results might reflect a bacterial strategy to limit the ability of the DC to increase MHC-II synthesis and thus possibly impair the presentation of relevant pathogen-derived peptides.

### *S. suis-*Stimulated DCs Upregulate Their Transcriptional Expression of MARCH1 and MARCH8, Both *In Vitro* and *In Vivo*, With the CPS Playing a Partial Role in This Regulation

To identify additional mechanisms potentially responsible for MHC-II modulation, the transcriptional expression of the ubiquitin ligases MARCH1 and MARCH8 was evaluated. As these molecules are involved in the fate (either lysosomal degradation or recycling) of pMHC-II at the cell surface, these molecules were promising regulatory candidates to investigate. BmDCs were stimulated *in vitro* with *S. suis* strains P1/7 and Δ*cpsF* for 1, 2, or 4 h. LPS-treated bmDCs showed *MARCH1* and *MARCH8* downregulation, as is expected in bmDCs responding to a TLR ligand. Meanwhile, transcription of these genes was poorly downregulated or even upregulated in *S. suis-*stimulated bmDCs (Figures 4B,C). In fact, incubation with the WT strain led to *MARCH1* levels significantly higher than those observed in LPS-treated bmDCs at all time points. As MARCH1 is known to ubiquitinate the costimulatory molecule CD86 as well as the MHC-II molecules, CD86 cell surface expression was analyzed by FACS to corroborate these results. *S. suis*-stimulated bmDCs displayed percentages of CD86^+^ cells that remained similar to those found in unstimulated cells, until at least 4 h, which is thus in agreement with the sustained/upregulated levels of *MARCH1*. Meanwhile, LPS-stimulated cells showed an increased expression of CD86 (Figure S5 in Supplementary Material). Similarly, the WT strain induced significantly higher *MARCH8* levels than those obtained with LPS at early time points in bmDCs. As for the role of the CPS, cells stimulated with the non-encapsulated mutant quickly downregulated *MARCH1* and *MARCH8*, and their expression levels became significantly different from those obtained with the WT strain at 2 h (Figures 4B,C). These results suggest that the presence of the CPS allows *S. suis* to hamper antigen presentation by promoting ubiquitination of MHC-II and thus lysosomal degradation of these molecules early after infection. *MARCH1* and *MARCH8* expression levels were also investigated *in vivo* at 3 and 6 h after infection. Splenic DCs derived from *S. suis-*infected mice showed sustained levels until at least 3 h for *MARCH1* and 6 h for *MARCH8* (Figures 5B,C). These results might support the previous *in vitro* findings or reflect just as well DC indirect activation.

### *S. suis* Infection Interferes With IL-12 Production in Splenic DCs but Does Not Impair Other Cytokine Production

**Figure 6:**
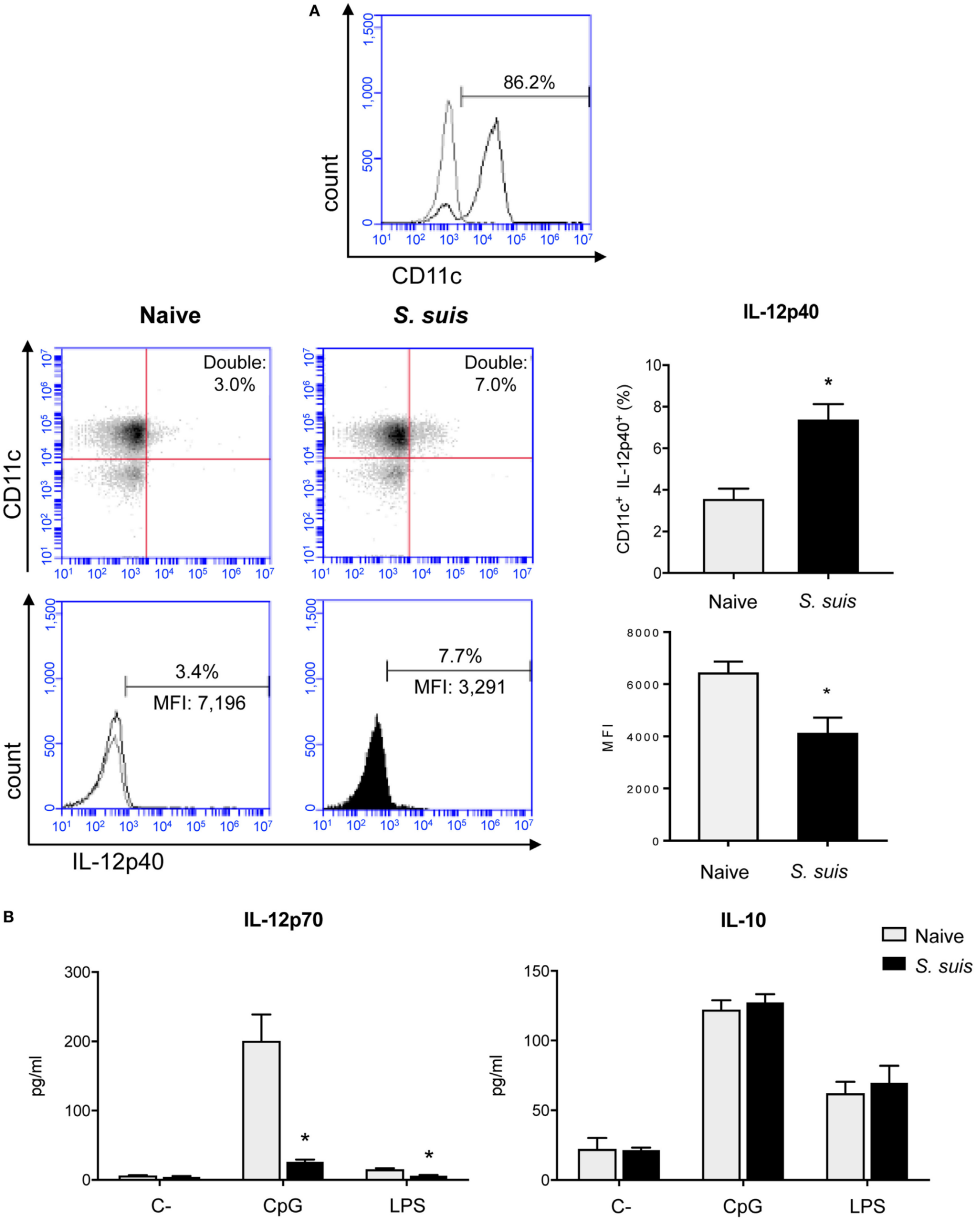
Splenic dendritic cells (DCs) have an impaired IL-12p70 secretion capacity following *Streptococcus suis* infection. C57BL/6 mice were injected intraperitoneally with 5 × 10^7^ CFU of *S. suis* wild-type (WT) strain P1/7. Spleens from mock-infected (naïve) and infected mice were harvested 6 h after infection (*n* = 8 per group). Splenic DCs were purified by MACS positive selection and CD11c purity is shown in **(A)**. Cells were stained for IC-FACS analysis of IL-12p40 expression. Events are gated on total cells and data are expressed as mean ± SEM [% of double positive cells or of mean fluorescence intensity (MFI)]. Representative dot plots and histograms have been selected for this figure. The gray line on the histogram is the isotype control. 30,000 gated events were acquired per sample and data analysis was performed using BD Accuri™ C6 software. Quadrants were drawn based on Alexa488- and APC-control stains and plotted on logarithmic scales. **(B)** Cells were cultured for 24 h *ex vivo* with CpG oligodeoxynucleotides (1 µM) or LPS (1 µg/ml). Supernatants were harvested and IL-12p70 and IL-10 levels were quantified by ELISA. Unstimulated cells served as negative controls (C−) for basal expression. Data are expressed as mean ± SEM (in pg/ml). *P < 0.05 indicates a statistically significant difference compared to naïve cells.

**Figure 7:**
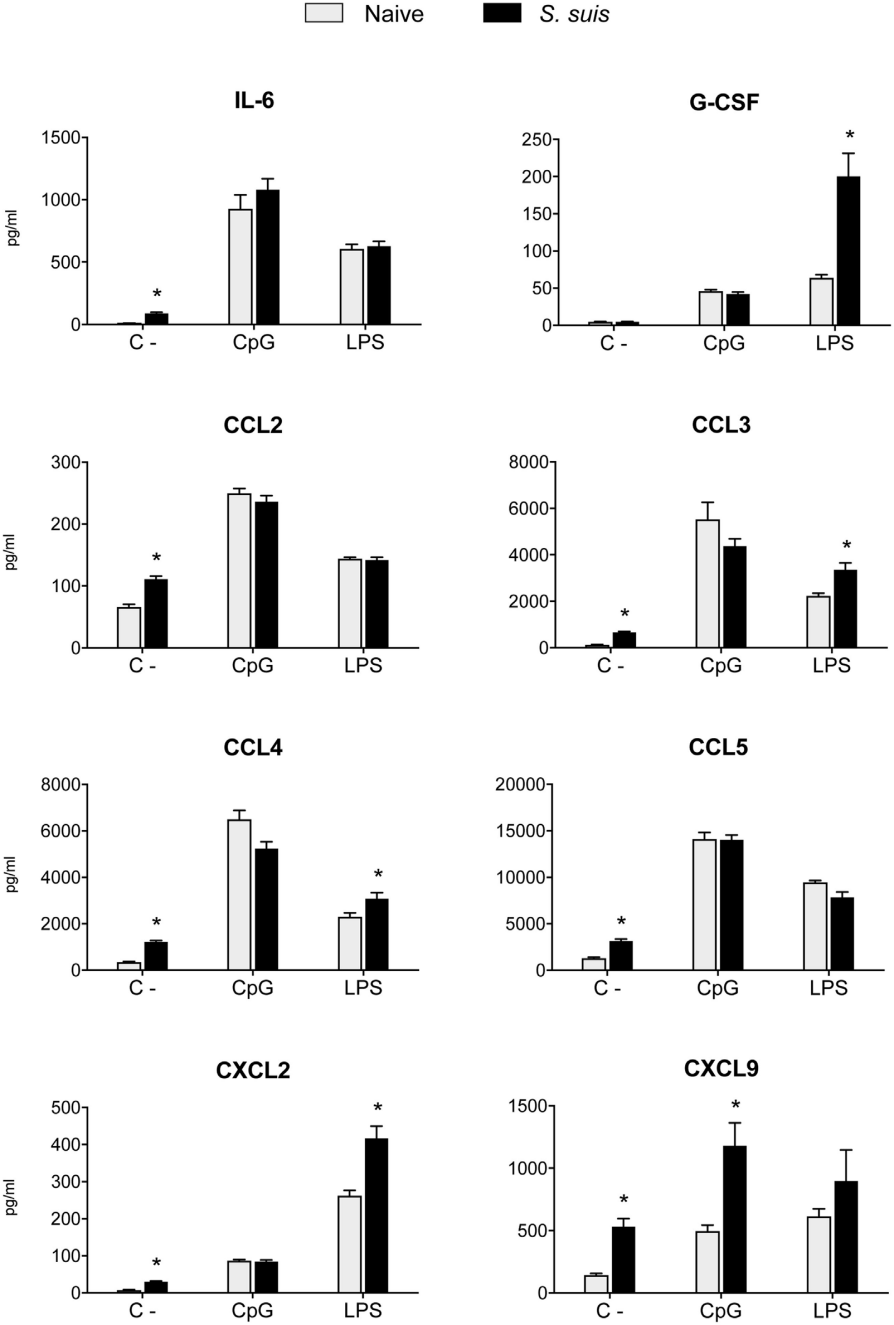
Splenic dendritic cells (DCs) maintain their secretion capacity of various cytokines and chemokines following *Streptococcus suis* infection. C57BL/6 mice were injected intraperitoneally with 5 × 10^7^ CFU of *S. suis* wild-type (WT) strain P1/7. Spleens from mock-infected (naïve) and infected mice were harvested 6 h after infection (*n* = 8 per group). Splenic DCs were purified by MACS positive selection. Cells were cultured for 24 h *ex vivo* with CpG oligodeoxynucleotides (1 µM) or LPS (1 µg/ml). Supernatants were harvested, and cytokine and chemokine levels were quantified by Luminex. Unstimulated cells served as negative controls (C−) for basal expression. Data were collected with Bio-Plex Manager™ software and analyzed with Bio-Plex^®^ MAGPIX system. Data are expressed as mean ± SEM (in pg/ml). *P < 0.05 indicates a statistically significant difference compared to naïve cells.

As Th1 immune responses are known to be protective against *S. suis* infection, the production of IL-12, the key Th1-polarizing signal 3, by splenic DCs was evaluated *in vivo* at 6 h after infection. The p40 subunit of IL-12 (also described as a subunit of IL-23) was detected by IC-FACS. Splenic DCs derived from *S. suis-*infected mice showed significantly higher percentages of IL-12p40^+^ cells than naïve controls, but their MFI levels were overall reduced (Figure 6A). To detect IL-12p70 (the bioactive heterodimeric form), splenic DCs from either naïve or infected mice were cultured *ex vivo* with CpG or LPS for 24 h and the supernatants were tested by ELISA. DCs from infected mice failed to produce IL-12p70 in both instances, while their naïve counterparts produced significant amounts of this cytokine (Figure 6B). Meanwhile, unstimulated DCs, derived from both naïve and infected mice, showed no significant production of IL-12p70. Interestingly, IL-10 production remained unchanged in DCs derived from *S. suis-*infected mice in response to CpG or LPS (Figure 6B). This thus led to an altered IL-10/IL-12 ratio, as reported previously in *S. suis-*stimulated bmDCs (29). To confirm that this inhibition was IL-12p70 specific and had no effect on cytokine production in a more general way, we also measured other cytokine levels by Luminex in these *ex vivo* supernatants. Following stimulation with CpG or LPS, DCs from infected mice were found to release similar or higher amounts of IL-6, G-CSF, CCL2, CCL3, CCL4, CCL5, CXCL2, and CXCL9, as compared to naïve DCs (Figure 7). Thus, mere cytotoxicity does not seem to be the underlying reason for DC impaired IL-12p70 production. In fact, these data rather suggest that DC exposure to inflammatory compounds during the acute phase of *S. suis* infection modulates the T cell-polarizing signal 3.

### *S. suis* Infection Impairs the MHC-II-Restricted Antigen Presentation Capacity of Splenic DCs

**Figure 8:**
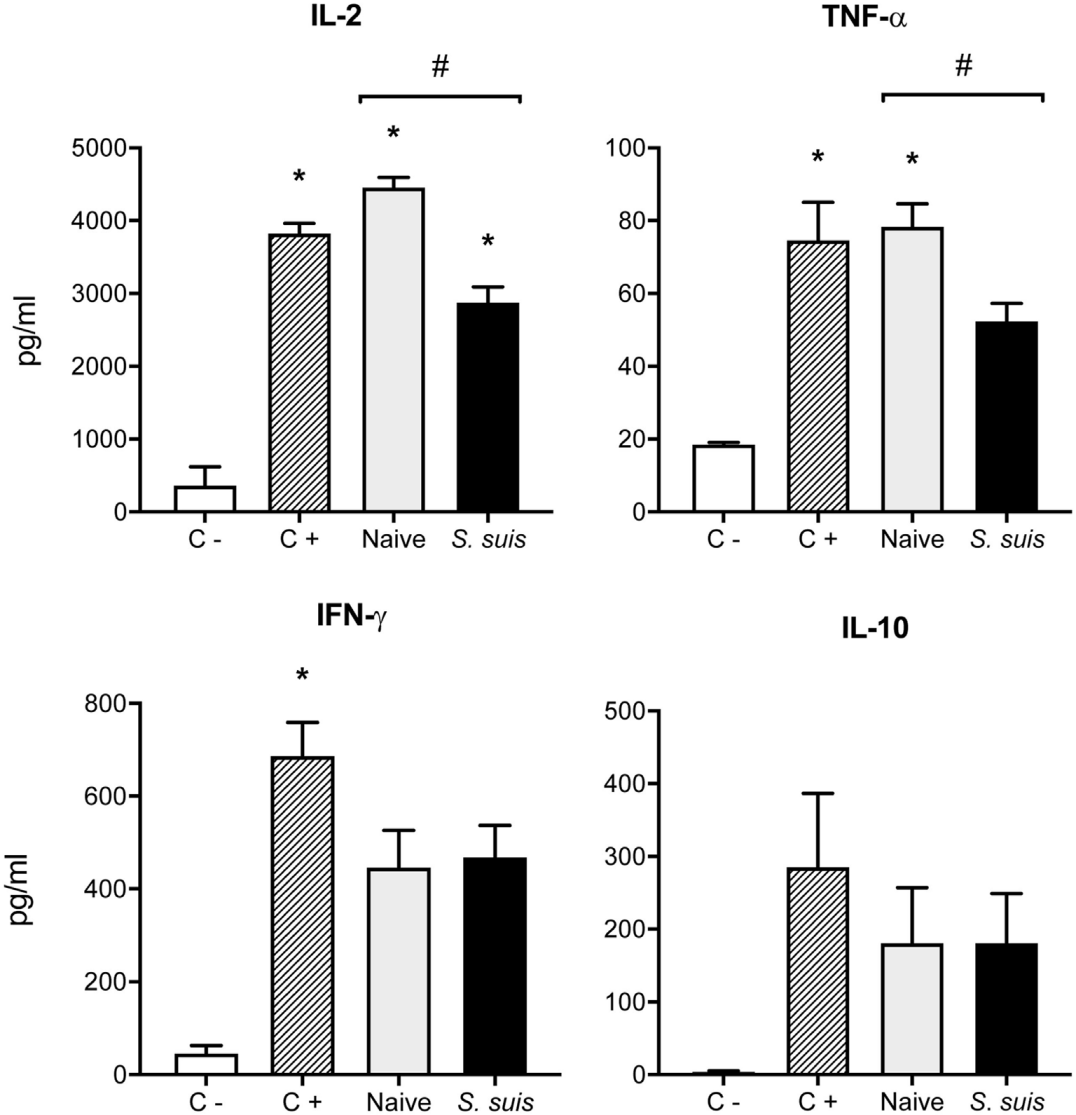
Splenic dendritic cells (DCs) from infected mice induce low Th1 cytokine production by CD4^+^ T cells. C57BL/6 mice were injected intraperitoneally with 5 × 10^7^ CFU of *Streptococcus suis* wild-type (WT) strain P1/7. Spleens from mock-infected (naïve) and infected mice were harvested 6 h after infection (*n* = 8 per group). Splenic DCs were purified by MACS positive selection and cocultured 24 h *ex vivo* with BO97.10 cells (DC:T cell ratio of 1:3) in Kappler–Marrack complete medium containing ovalbumin (OVA) (500 µg/ml) and antibiotics (100 µg/ml gentamycin). Supernatants were harvested and IL-2, TNF-α, IFN-γ, and IL-10 levels were quantified by ELISA. Cocultures incubated with medium alone served as negative controls (C−), while cocultures treated with LPS (1 µg/ml) and OVA served as positive controls (C+). Data are expressed as mean ± SEM (in pg/ml). *P < 0.05 indicates a statistically significant difference compared to unstimulated control cells. ^#^*P* < 0.05 indicates a statistically significant difference between cells derived from naïve and infected mice.

**Figure 9:**
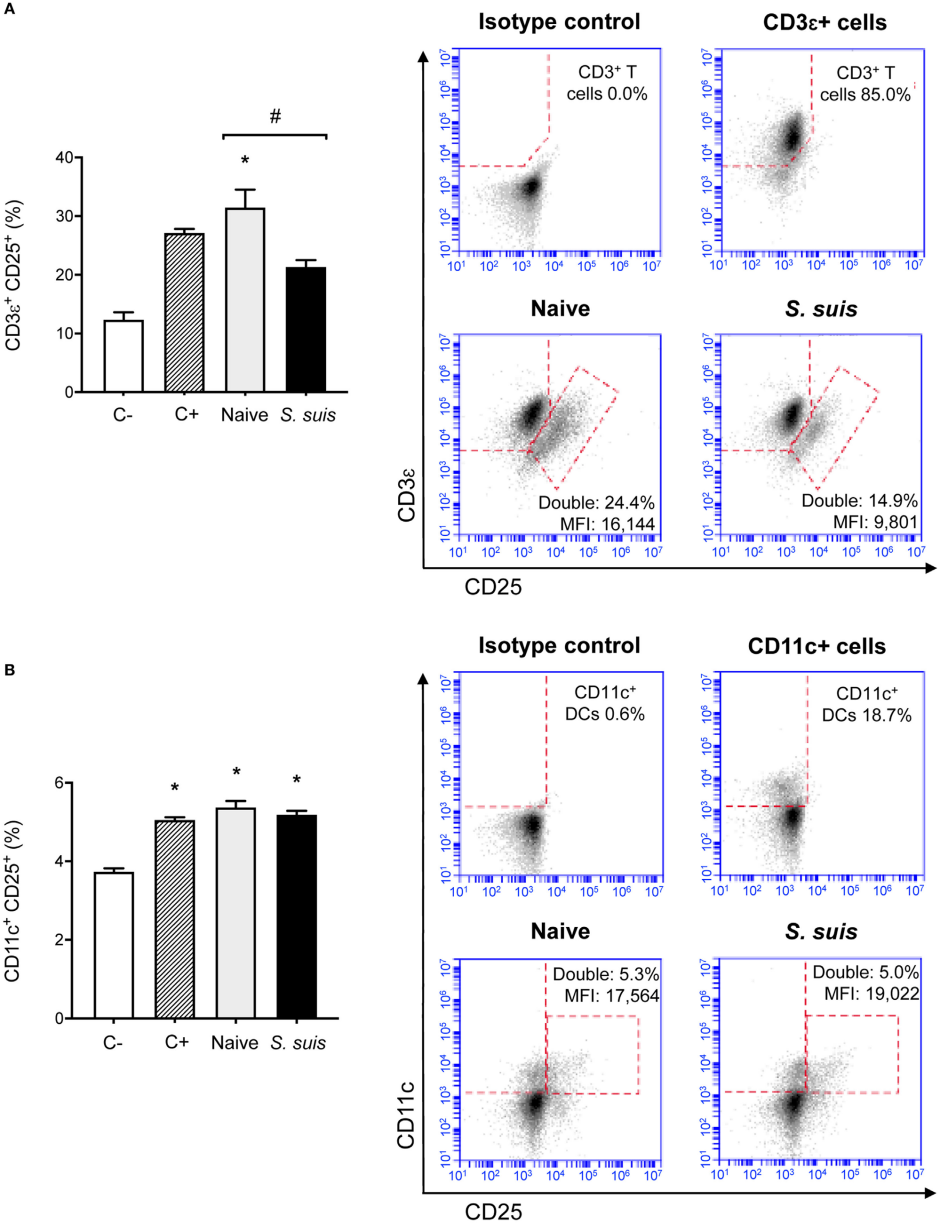
Splenic dendritic cells (DCs) from infected mice induce low CD25 expression on CD3ε^+^ cells but not on CD11c^+^ cells. C57BL/6 mice were injected intraperitoneally with 5 × 10^7^ CFU of *Streptococcus suis* wild-type (WT) strain P1/7. Spleens from mock-infected (naïve) and infected mice were harvested 6 h after infection (*n* = 8 per group). Splenic DCs were purified by MACS positive selection and cocultured 24 h *ex vivo* with BO97.10 cells (DC:T cell ratio of 1:3) in Kappler–Marrack complete medium containing ovalbumin (OVA) (500 µg/ml) and antibiotics (100 µg/ml gentamycin). Cocultures incubated with medium alone served as negative controls (C−), while cocultures treated with LPS (1 µg/ml) and OVA served as positive controls (C+). Cells were harvested and surface stained for FACS analysis of CD25 expression on **(A)** CD3ε^+^ or **(B)** CD11c^+^ cells. Events are gated on total cells and data are expressed as mean ± SEM (% of double positive cells). Representative dot plots have been selected for this figure, including mean fluorescence intensity (MFI) values. 30,000 gated events were acquired per sample and data analysis was performed using BD Accuri™ C6 software. Quadrants were drawn based on FITC-, PE/Cy5-, and APC-control stains and plotted on logarithmic scales. *P < 0.05 indicates a statistically significant difference compared to unstimulated control cells. ^#^*P* < 0.05 indicates a statistically significant difference between cells derived from naïve and infected mice.

Finally, we analyzed whether *S. suis* infection could interfere with MHC-II-restricted antigen presentation. To this aim, we cocultured for 24 h splenic DCs derived from either naïve or infected mice with the T cell hybridoma BO97.10, specific for OVA_323–339_ epitope on I-A^b^. The production of IL-2, TNF-α, IFN-γ, and IL-10 was measured in the supernatants by ELISA, as a way to evaluate CD4^+^ T cell activation in response to processing and presentation of the OVA_323–339_ peptide by DCs. CD4^+^ T cells incubated with DCs from infected mice showed significantly lower IL-2 production than CD4^+^ T cells exposed to naïve DCs (Figure 8). This diminished CD4^+^ T cell response was further supported by the reduced expression (at both % and MFI levels) of the CD25 receptor (IL-2 receptor α chain) that was observed by FACS on CD3ε^+^ cells after 24 h of coculture with DCs from infected mice. Meanwhile, CD25 expression remained unchanged on CD11c^+^ cells (Figure 9). DCs derived from *S. suis-*infected mice also induced a lower TNF-α production by CD4^+^ T cells as compared to their naïve counterparts (Figure 8). While *S. suis* infection did not alter the OVA-induced production of IFN-γ, it is interesting to note that these cells also failed to upregulate their IFN-γ production to the levels obtained with the positive control. No increased production of the regulatory cytokine IL-10 was detected in the cocultures, as was observed with the splenic DCs cultured *ex vivo* (Figure 6B).

Single cell cultures (either DCs or T cells alone) were included as controls and showed no significant cytokine production. Moreover, the WT strain ability to induce polyclonal CD4^+^ T cell activation was investigated in *S. suis*-infected cocultures without OVA and was found to be negligible (data not shown). Interestingly, bmDCs infected *in vitro* with *S. suis* (thus yielding dir-mDCs) and cocultured with BO97.10 cells induced no clear difference in OVA-induced IL-2 and TNF-α levels, compared to non-infected cocultures (Figure S1 in Supplementary Material). Hence, these results suggest that indirect activation of DCs during the acute, pro-inflammatory phase of *S. suis* infection leads to suboptimal CD4^+^ T cell activation, while direct activation of DCs by *S. suis* itself might not interfere with this process to the same extent.

## Discussion

While *S. suis* is known as a potent inducer of pro-inflammatory mediators during acute/peracute systemic infections (3, 6, 28), evidence of its ability to impair various immune cell functions and prevent the development of an effective adaptive immune response is not less substantial (6, 29, 32). In this regard, *S. suis* modulations of DC functions have been documented in mice, swine, and humans (9, 28–30), and CD4^+^ T cells were shown to be poorly activated during *S. suis* infection (32). However, a comprehensive evaluation of the DC antigen presentation ability has yet to be performed. This prompted us to evaluate MHC-II-restricted antigen presentation by DCs, as well as the other signals required for CD4^+^ T cell activation.

T cell receptor recognition of a specific antigenic peptide presented on an MHC-II molecule constitutes the first signal for CD4^+^ T cell activation. In order for an extracellular pathogen such as *S. suis* to have its antigenic peptides enter the MHC-II pathway in a DC, it must first be captured and processed by the APC. Therefore, we investigated the ability of *S. suis-*stimulated bmDCs to capture and degrade a soluble protein into peptides, using DQ-OVA. The CPS of *S. suis* is well known for conferring resistance to phagocytosis (7, 29, 38), and it has been shown to inhibit the entry of latex beads in macrophages (7); we thus expected *S. suis*-stimulated DCs to display impaired capture and consequently process OVA. However, our observations led us to conclude that, in DCs, *S. suis* does not significantly affect this phase of antigen presentation. *S. suis* CPS has been reported to block phagocytosis of bacterial size particles (such as latex beads), yet other endocytic pathways might remain unaltered. In fact, macropinocytosis has been described as a far more important endocytic process in DC-mediated antigen presentation to T cells (8). It should also be noticed that once *S. suis* is internalized (albeit at low levels) (9, 39), it is rapidly degraded intracellularly, thereby supporting the results of normal processing of DQ-OVA.

The MHC-II pathway involves a series of complex intracellular events that eventually lead to the trafficking of pMHC-II to the cell surface. The expression of MHC-II molecules on *S. suis-*stimulated DCs had only been studied thus far at long incubation times (16 h), like a mere cellular maturation marker (9, 29). However, the events that take place in the first few hours following stimulation appear to be decisive for the antigen repertoire selection, at least for dir-mDCs as these cells process microbial antigens at the time of the TLR stimulus only (27). Hence, we sought to investigate MHC-II expression in DCs responding to *S. suis* at short incubation times. Unfortunately, due to the lack of immunological tools available for *S. suis*, loading of MHC-II molecules with *S. suis*-derived peptides could not be evaluated directly in the present study, and the expression of MHC-II molecules on the cell surface (e.g., independently of the antigenic fragments they bore) was investigated instead. Through comparison with LPS-treated cells, *in vitro* kinetic studies evidenced a delayed increase of MHC-II^+^ cells in *S. suis-*stimulated bmDCs. Interestingly, this delay appeared to be more pronounced with the encapsulated strain, suggesting a role of the CPS in this modulation by either limiting bacterial internalization and/or hindering the recognition of immunogenic cell wall components by TLRs at the cell surface level (9, 29). We cannot undermine here the limitations of comparing *S. suis* with a pure TLR4 ligand, as a live pathogen is bound to interact with DCs in a much more complex way than the latter. However, for the sake of comparison with literature, LPS offered interesting perspectives: this ligand has been used extensively in molecular studies of the MHC-II pathway (in contrast to TLR2 ligands) and has also proven to be useful in the context of *S. suis* for normalizing the expression of DC maturation markers (30). The reduction of the MHC-II expression levels on naïve splenic DCs stimulated *in vitro* with encapsulated *S. suis* or splenic DCs derived from *S. suis-*infected mice supports the relevance of the *in vitro* findings and is consistent with a modulation of MHC-II expression by encapsulated *S. suis*. Moreover, it has been reported that the timing of DC antigen encounter and the subsequent changes induced by DC maturation can dramatically affect the outcome of vaccination (10).

To get an insight into the mechanisms potentially involved in the modulation of MHC-II expression in *S. suis*-stimulated DCs, transcriptional expression of CIITA, MARCH1, and MARCH8 was evaluated. Low *CIITA* mRNA levels were observed in *S. suis-*stimulated bmDCs and splenic DCs from *S. suis-*infected mice. These results suggest that *S. suis* impairs the ability of DCs to increase the synthesis of new MHC-II molecules shortly after they encounter the pathogen. The fact that newly synthesized MHC-II molecules constitute the primary source for antigen presentation (10) underscores the likelihood that *S. suis* disturbs the optimal timing between the processing of pathogen-derived antigens and MHC-II loading/trafficking, through *CIITA* modulation. Similarly, monocytes/macrophages infected with *Mycobacterium tuberculosis* or *M. bovis* BCG (40, 41) or *Brucella abortus* (42) were shown to downregulate the expression of IFN-γ-stimulated *CIITA* and MHC-II genes at very early time points in a way to prevent recognition by T cells and establish a chronic infection. Yet, this is the first time that *CIITA* regulation is observed in DCs with an extracellular pathogen. As for the transcription of the ubiquitin ligases MARCH1/8, our results suggest that the encapsulated strain of *S. suis* hijacks MARCH1/8-mediated MHC-II ubiquitination in DCs, thereby promoting lysosomal degradation of these molecules with obvious consequences on antigen presentation to CD4^+^ T cells. Modulation of *MARCH1* expression and subsequent MHC-II downregulation has already been described in *Francisella tularensis*-infected macrophages and attributed to a PGE_2_-inducible host factor capable of inducing IL-10 (43). However, it appears unlikely that *S. suis* would use a similar mechanism to modulate *MARCH1* expression in DCs since the effect of IL-10 on antigen presentation is MARCH1-independent in this cell type (11). Our *in vivo* results support, at least in part, a modulation of *MARCH1/8* expression by *S. suis*, as sustained transcription levels of these molecules were observed in splenic DCs derived from infected mice, without involving IL-10 upregulation. In a murine model of multiple organ dysfunction syndrome (MODS), which is characterized by the loss of control over systemic inflammatory responses, the protein expression levels of MHC-II and functional immune activities of DCs were also inversely related to *MARCH1* expression. The authors suggested that MARCH1-mediated ubiquitination and aberrant degradation of MHC-II on DC surfaces affected the pathological progression of MODS (44).

Modulation of MHC-II expression in *S. suis-*stimulated DCs cannot be analyzed without discussing the maturation profile of the cells. Although a clear and unanimous definition of mature and immunogenic DCs is yet to be determined, phenotypically mature DCs are usually defined, regardless of their activation mode, by their high surface expression of appropriate “maturation markers” such as MHC-II, CD80, CD86, CD40, and CCR7 (20, 21). BmDCs were most probably activated directly in our *in vitro* experiments, as autocrine production of inflammatory mediators is likely negligible in a closed system at short incubation times. However, the delayed MHC-II kinetics and a low CD86 expression that we observed at early time points suggest that *S. suis* failed to induce optimal cellular maturation at those times. Upregulation of *MARCH1/8* in *S. suis-*stimulated bmDCs could be responsible for their impaired MHC-II and CD86 expression, but other regulatory mechanisms must be involved, as bmDCs stimulated with the non-encapsulated mutant still displayed a low CD86 expression despite an adequate downregulation of *MARCH1/8*. Meanwhile, splenic DCs from infected mice showed high percentages of CD86^+^ cells but low *CIITA* expression and sustained mRNA levels of *MARCH1/8*, with concomitant reduction in expression levels of MHC-II. These last results support our *in vitro* findings, even though splenic DCs form a heterogeneous population of DC subsets with distinct maturation phenotypes, levels of infection, and T cell-priming capacities (45). *S. suis* could also interact differently with DCs of various origins, as *in vitro*-stimulated bmDCs and splenic DCs were found to differ in their MHC-II expression profile at late time points. Finally, our *in vivo* approach possibly yielded both dir-mDCs and indir-mDCs as splenocytes had the opportunity to encounter either the pathogen itself or the inflammatory mediators released by innate immune cells during the acute phase of the infection.

The last signal required for full CD4^+^ T cell activation is the production of polarizing cytokines by the APC. Host protection against *S. suis* is highly dependent on antibodies associated with Th1 immune responses (2). However, encapsulated *S. suis* serotype 2 has been shown to induce, in murine and human DCs *in vitro*, a high IL-10/IL-12 ratio, which indicates the potential to polarize T cell responses toward Th2/Treg, or at least to impair optimal T cell activation (29, 30). Actually, *S. suis* systemic infection induces a weak Th1 response with low levels of TNF-α, IFN-γ, and IL-2 as well as the production of IL-10. *S. suis* CPS is known to interfere with CD4^+^ T cell activation (32). Here, we found that splenic DCs derived from *S. suis-*infected mice specifically failed to secrete IL-12p70 in response to *ex vivo* stimulation with CpG or LPS, thus revealing that the infection primarily leads, in fact, to indir-mDCs. Consequently, these cells elicited a significant inhibition of IL-2 production by CD4^+^ T cells in an antigen presentation assay, along with low levels of TNF-α and IFN-γ. These results were further supported by the downregulated expression of CD25 that we observed on CD3ε^+^ cells, which reflects the inhibition of T cell activation (46) in response to *S. suis* infection. Meanwhile, the fact that CD11c^+^ cells from infected mice expressed similar levels of CD25 as their naïve counterparts might suggest that the infection does not induce regulatory DCs (47, 48) or Th17-polarizing DCs (49). Nevertheless, the slight increase of IL-12p40^+^ cells that we detected by IC-FACS in splenic DCs from infected mice might still reflect the initiation of a Th17 response—rather than representing a bioactive IL-12p70 production—as IL-12p70 shares its subunit p40 with IL-23, a key cytokine for Th17 and Th1 responses against extracellular bacteria (19, 50). Interestingly, the direct activation of porcine or mouse bmDCs by *S. suis* results in increased levels of IL-23 production *in vitro* (29, 51), once again suggesting possible generation of both dir-mDCs and indir-mDCs during *in vivo S. suis* infection.

Similarly, *M. tuberculosis* has been reported to impair IL-12p70 secretion and antigen presentation to CD4^+^ T cells, thus diverting T cell differentiation toward Tregs or Th2 cells that are not productive toward an intracellular pathogen like *M. tuberculosis* (52, 53). Impaired antigen presentation ability of DCs has also been demonstrated for *Salmonella enterica* (54, 55) and *B. suis* (56), all intracellular pathogens. Albeit these mechanisms seem to be generalized to several intracellular pathogens, this is the first time that *in vitro* and *in vivo* transcriptional regulation of MHC-II, *ex vivo* DC antigen presentation, and downstream effects on T cell activation are evaluated in the context of an infection with an extracellular pathogen. To the best of our knowledge, only one study has previously reported that bmDCs exposed *in vitro* to live *S. mutans* fail to drive antigen-specific T cell proliferation (57), which is different from what was observed with *S. suis* dir-mDCs in this study. Finally, *in vivo* experiments conducted in parallel with another Gram-positive encapsulated bacterium, Group B *Streptococcus* (GBS), have shown that both pathogens have similar surface expression profiles of MHC-II and CD86 and transcription levels of antigen presentation genes (unpublished observations); yet, GBS-infected mice developed optimal primary and memory T cell responses (58). It thus remains to further investigate whether the observed modulation of IL-12p70 is a feature common to systemic inflammatory response-inducing agents, a particular bacterial immune evasion strategy, or even a combination of both mechanisms.

This work offers a comprehensive study of the signals involved in antigen presentation in DCs responding to *S. suis* and their consequences on CD4^+^ T cell activation. We have shown that *S. suis* modulates MHC-II expression, both *in vitro* and *in vivo*. *S. suis* CPS was found to be only partly accountable for these transcriptional profiles. In fact, a variety of factors defining the strain pathogenicity might be involved in the modulation of the APC functions by systemic inflammatory response-inducing pathogens. Nevertheless, this is the first report of an *in vivo* transcriptional kinetics study of CIITA and MARCH1/8 in DCs responding to a live bacterial pathogen. More importantly, we described, in splenic DCs from infected mice, a cytokine secretion profile that is compatible with indirect activation by inflammatory mediators during the acute phase of *S. suis* infection. These cells have an impaired MHC-II-restricted antigen presentation capacity and an altered cytokine profile, which could lead to the formation of heterogeneous Th cells (21). This study thus highlights the potential consequences of inflammation on the type and magnitude of the immune response elicited by a pathogen. Better characterization of dir-mDCs and indir-mDCs profiles will help understand in the future how pathogens modulate T cell activation signals 1 and 2.

## Ethics Statement

This study was carried out in accordance with the recommendations of the guidelines and policies of the Canadian Council on Animal Care and the principles set forth in the Guide for the Care and Use of Laboratory Animals. The protocols and procedures were approved by the Animal Welfare Committee of the University of Montreal (protocol number rech-1399).

## Author Contributions

Conceived and designed the experiments: CL, MG, JT, and MS. Performed the experiments: CL, PL, J-PA, and TG. Analyzed the data and contributed to the writing of the manuscript: CL, MG, JT, and MS. All authors have read and approved the manuscript.

## Conflict of Interest Statement

The authors declare that the research was conducted in the absence of any commercial or financial relationships that could be construed as a potential conflict of interest.

## Abbreviations

bmDC, bone marrow-derived dendritic cell; CIITA, Class II major histocompatibility complex transactivator; CPS, capsular polysaccharide; dir-mDC, directly activated mature DC; indir-mDC, indirectly activated mature DC; MARCH, membrane-associated RING-CH; OVA, ovalbumin; pMHC-II, peptide–MHC-II complexes; WT, wild type.
