# Supplementary material for: Streptococcus suis Serotype 2 Infection Impairs Interleukin-12 Production and the MHC-II-Restricted Antigen Presentation Capacity of Dendritic Cells
Source: Front Immunol. 2018 May 30;9:1199. doi: 10.3389/fimmu.2018.01199 (PMC5988873; doi:10.3389/fimmu.2018.01199)
Supplement: Supplementary file 1 [file Presentation_1.pdf]

Supplementary Material

Streptococcus suis Serotype 2 Infection Impairs IL-12 Production and the MHC-II-Restricted Antigen Presentation Capacity of Dendritic Cells

Corinne Letendre, Jean-Philippe Auger, Paul Lemire, Tristan Galbas, Marcelo Gottschalk, Jacques Thibodeau*, and Mariela Segura*

***Correspondence (co-corresponding authors):**
Mariela Segura: mariela.segura@umontreal.ca
Jacques Thibodeau: jacques.thibodeau@umontreal.ca

Supplementary Figures

Figure S1. *S. suis*-stimulated bmDCs preserve their capacity to induce CD4^{+} T cell activation.
Figure S2. *S. suis*-stimulated bmDCs preserve their antigen capture and processing capacities.
Figure S3. BmDCs enhance their MHC-II cell surface expression with a delayed kinetics upon stimulation with *S. suis*.
Figure S4. Naïve splenic DCs show low intensity of MHC-II expression upon *in vitro* stimulation at short incubation times with the encapsulated strain of *S. suis*.
Figure S5. BmDCs do not enhance their CD86 cell surface expression at short incubation time points following stimulation with *S. suis*.

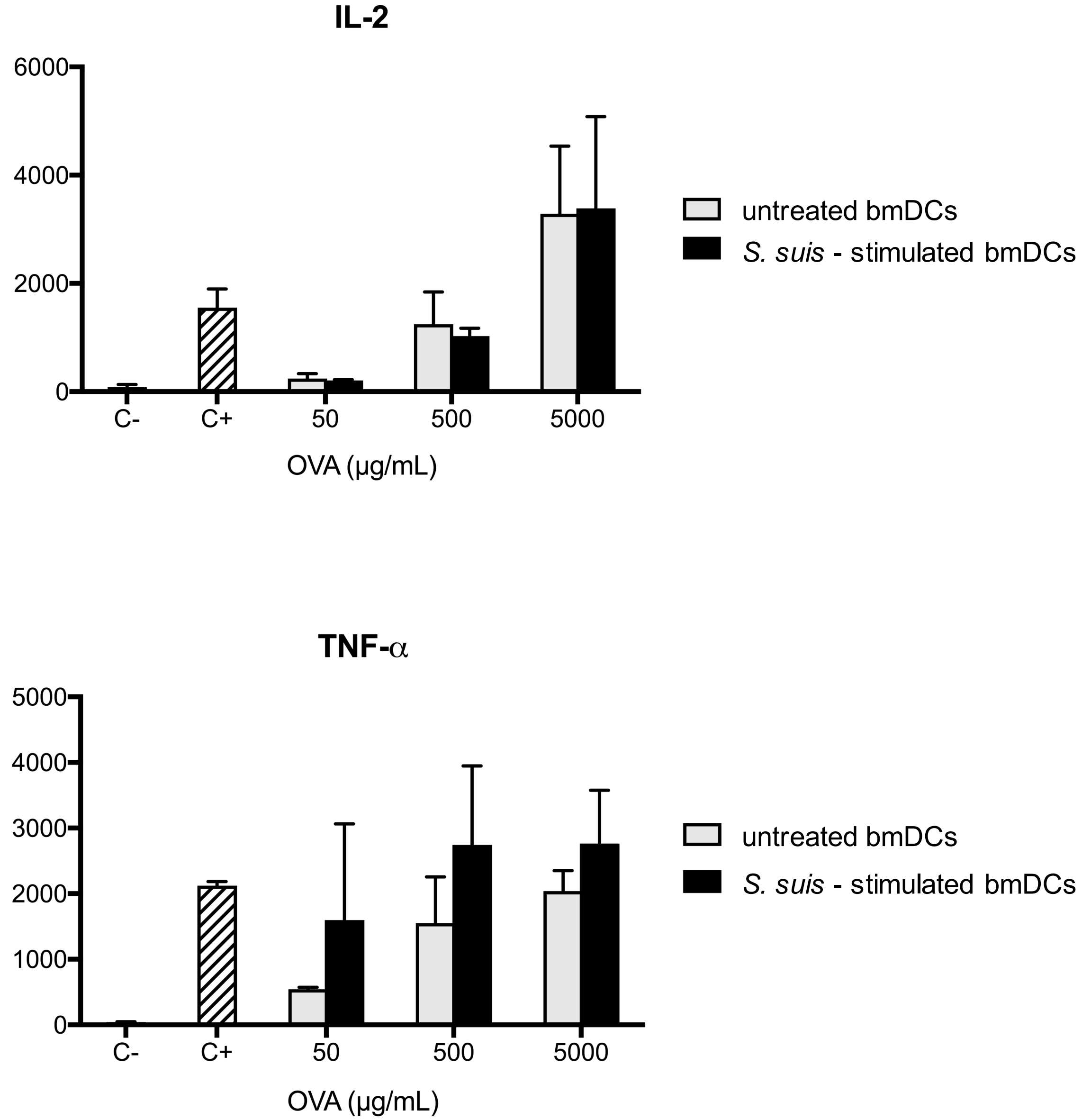

Supplementary Figure S1. *S. suis*-stimulated bmDCs preserve their capacity to induce CD4^{+} T cell activation. Cells were stimulated for 2 h with *S. suis* wild-type (WT) strain P1/7 in RPMI supplemented medium containing ovalbumin (OVA: 50, 500, or 5000 µg/ml). BmDCs were then cocultured for 24 h with BO97.10 cells (DC: T cell ratio of 1: 3) in Kappler Marrack complete medium without antibiotics to allow initial bacterial multiplication (100 µg/ml gentamycin added after 14 h to prevent cell toxicity). Supernatants were harvested and IL-2 and TNF-α levels were quantified by ELISA. Cocultures incubated with medium alone served as negative controls (C-), while cocultures treated with LPS (1 µg/ml) and OVA served as positive controls (C+). Data are expressed as mean ± SEM (in pg/ml). $^{*}P < 0.05$, indicates a statistically significant difference compared to control (untreated) bmDCs.

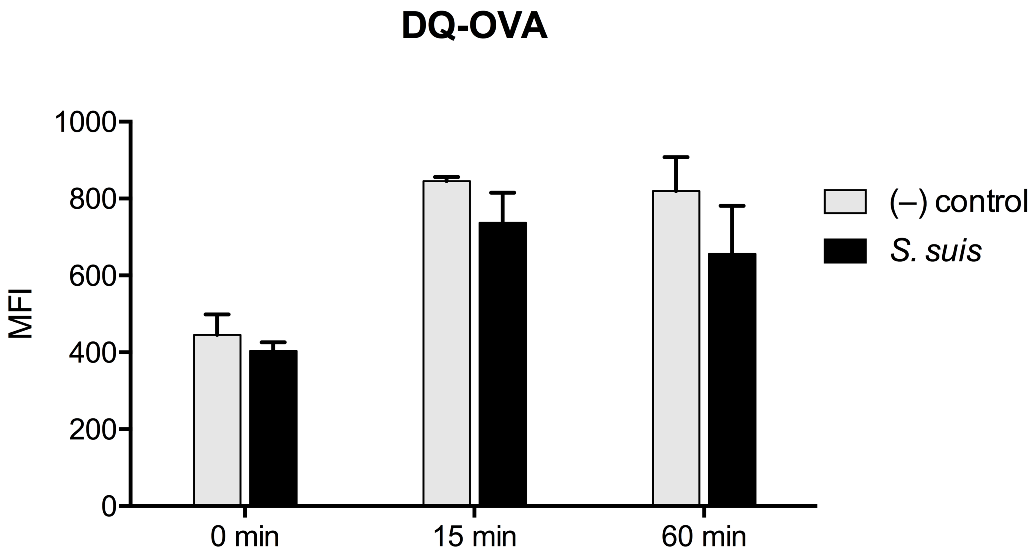

Supplementary Figure S2. *S. suis*-stimulated bmDCs preserve their antigen capture and processing capacities. Cells were stimulated for 45 min with *S. suis* wild-type (WT) strain P1/7 (initial MOI:10) prior to incubation with DQ-OVA (50 µg/ml) or with non-labeled OVA for 15 min at 37˚C. After washing steps to remove non-internalized OVA, cells were then incubated for the indicated times (0 to 60 min) and assayed by FACS to evaluate processing of OVA into peptides after internalization. Non-infected cells served as negative (–) control. Data are expressed as the increase in mean fluorescence intensity (MFI) over time ± SEM and are from 2 independent experiments. Twenty thousand gated events were acquired per sample and data analysis was performed using Cell Lab Quanta Collection/Analysis software.

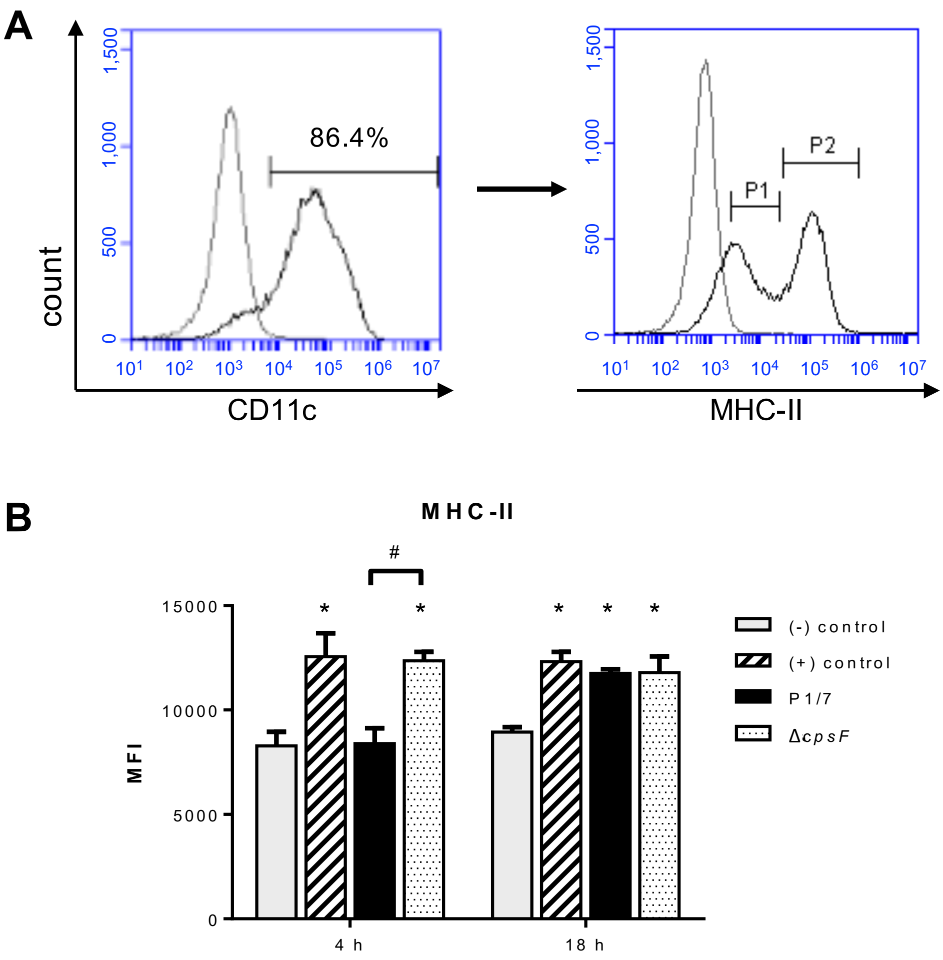

Supplementary Figure S3. BmDCs enhance their MHC-II cell surface expression with a delayed kinetics upon stimulation with *S. suis*. Cells were stimulated for 4 h or 18 h with *S. suis* wild-type (WT) strain P1/7 or the non-encapsulated mutant Δ*cpsF* (initial MOI:1) in technical duplicates. Unstimulated cells served as negative (–) control for basal expression at each time point. Cells stimulated with LPS (1 µg/ml) were used as positive (+) control. Cells were harvested and fixed after each incubation time. Once the last incubation time was over, cells were surface stained for CD11c and MHC-II, and analyzed by FACS. Events are gated on $CD11c^+$ cells. (**A**) Representative histograms have been selected for this figure (at time = 4 h) showing the P1 ($MHC\text{-}II^{low}$) and the P2 ($MHC\text{-}II^{high}$) subpopulations. The gray lines are isotype controls. At least thirty thousand gated events were acquired per sample and data analysis was performed using BD Accuri™ C6 software. (**B**) Data are expressed as mean ± SEM (in MFI levels for the **P1** subpopulation) and are from 3 independent experiments. $^*P < 0.05$ indicates a statistically significant difference compared to (–) control cells. $\#P < 0.05$ indicates a statistically significant difference between P1/7-stimulated bmDCs and Δ*cpsF*-stimulated bmDCs.

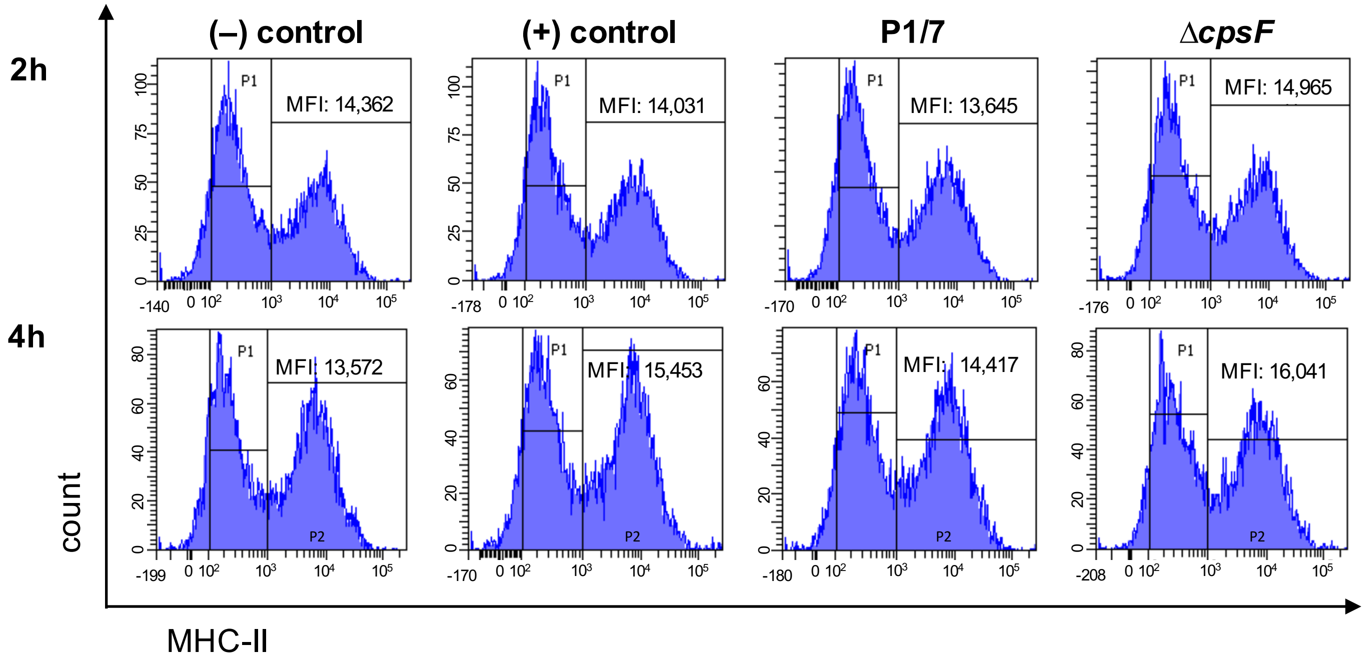

Supplementary Figure S4. Naïve splenic DCs show low intensity of MHC-II expression upon *in vitro* stimulation at short incubation times with the encapsulated strain of *S. suis*. Spleen cells from a pool of 10 naive C57BL/6 mice were FACS-purified and CD11c^{+} cells (APC-conjugated anti-CD11c) were sorted with the BD FACSAria^{TM} Fusion flow sorter. Purified cells were stimulated for 2 or 4 h with *S. suis* wild-type (WT) strain P1/7 or the non-encapsulated mutant Δ*cpsF* (initial MOI:1). Unstimulated cells served as negative (–) control for basal expression at each time point. Cells stimulated with LPS (1 µg/ml) were used as positive (+) control. Cells were harvested and surfaced stained for MHC-II. Histograms illustrate variations in the MFI of the MHC-II^{high} population (P2, gated on CD11c^{+} cells). Data analysis was performed using BD FACSDiva software.

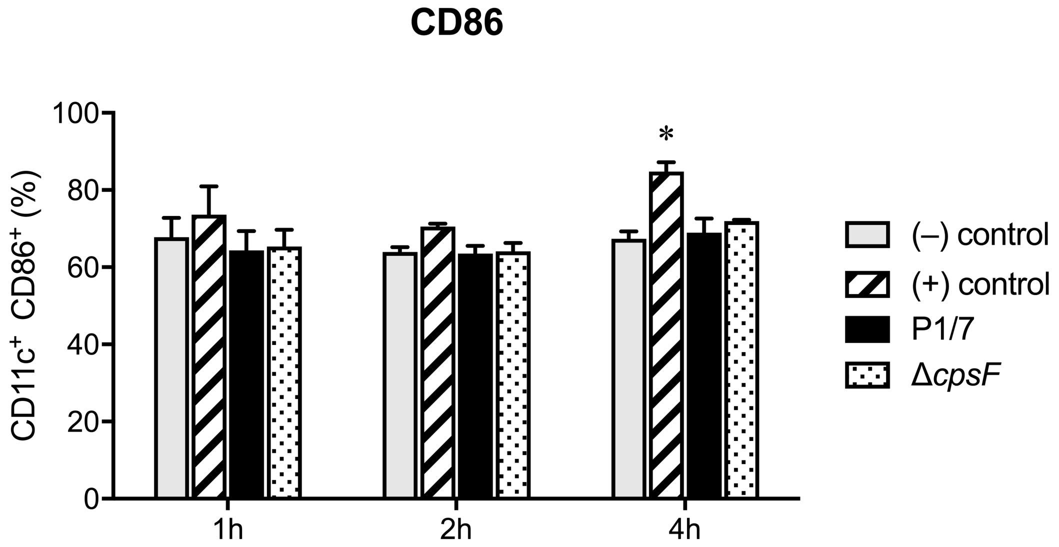

Supplementary Figure S5. BmDCs do not enhance their CD86 cell surface expression at short incubation time points following stimulation with *S. suis*. Cells were stimulated for 1, 2 or 4 h with *S. suis* wild-type (WT) strain P1/7 or the non-encapsulated mutant Δ*cpsF* (initial MOI:1). Cells were harvested and fixed after each incubation time. Once the last incubation time was over, cells were surface stained for CD11c and CD86, and analyzed by FACS. Unstimulated cells served as negative (–) control for basal expression at each time point. Cells stimulated with LPS (1 μg/ml) were used as positive (+) control. Data are expressed as mean ± SEM (in % of positive cells) from 2 independent experiments. At least thirty thousand gated events were acquired per sample and data analysis was performed using BD Accuri™ C6 software. Quadrants were drawn based on PE/Cy5- and PE-control and were plotted on logarithmic scales. $^{*}P < 0.05$, indicates a statistically significant difference compared to control cells.
